# Transcriptional and Epigenetic Substrates of Methamphetamine Addiction and Withdrawal: Evidence from a Long-Access Self-Administration Model in the Rat

**DOI:** 10.1007/s12035-014-8776-8

**Published:** 2014-06-18

**Authors:** Jean Lud Cadet, Christie Brannock, Subramaniam Jayanthi, Irina N. Krasnova

**Affiliations:** Molecular Neuropsychiatry Research Branch, Intramural Research Program, National Institute on Drug Abuse, NIH, DHHS, 251 Bayview Boulevard, Baltimore, MD 21224 USA

**Keywords:** Gene expression, Gene networks, Transcription factors, Epigenetics, HDAC, Repressor complexes, Cognition, Striatum

## Abstract

Methamphetamine use disorder is a chronic neuropsychiatric disorder characterized by recurrent binge episodes, intervals of abstinence, and relapses to drug use. Humans addicted to methamphetamine experience various degrees of cognitive deficits and other neurological abnormalities that complicate their activities of daily living and their participation in treatment programs. Importantly, models of methamphetamine addiction in rodents have shown that animals will readily learn to give themselves methamphetamine. Rats also accelerate their intake over time. Microarray studies have also shown that methamphetamine taking is associated with major transcriptional changes in the striatum measured within a short or longer time after cessation of drug taking. After a 2-h withdrawal time, there was increased expression of genes that participate in transcription regulation. These included cyclic AMP response element binding (CREB), ETS domain-containing protein (ELK1), and members of the FOS family of transcription factors. Other genes of interest include brain-derived neurotrophic factor (BDNF), tyrosine kinase receptor, type 2 (TrkB), and synaptophysin. Methamphetamine-induced transcription was found to be regulated via phosphorylated CREB-dependent events. After a 30-day withdrawal from methamphetamine self-administration, however, there was mostly decreased expression of transcription factors including junD. There was also downregulation of genes whose protein products are constituents of chromatin-remodeling complexes. Altogether, these genome-wide results show that methamphetamine abuse might be associated with altered regulation of a diversity of gene networks that impact cellular and synaptic functions. These transcriptional changes might serve as triggers for the neuropsychiatric presentations of humans who abuse this drug. Better understanding of the way that gene products interact to cause methamphetamine addiction will help to develop better pharmacological treatment of methamphetamine addicts.

## Introduction

**Fig. 1:**
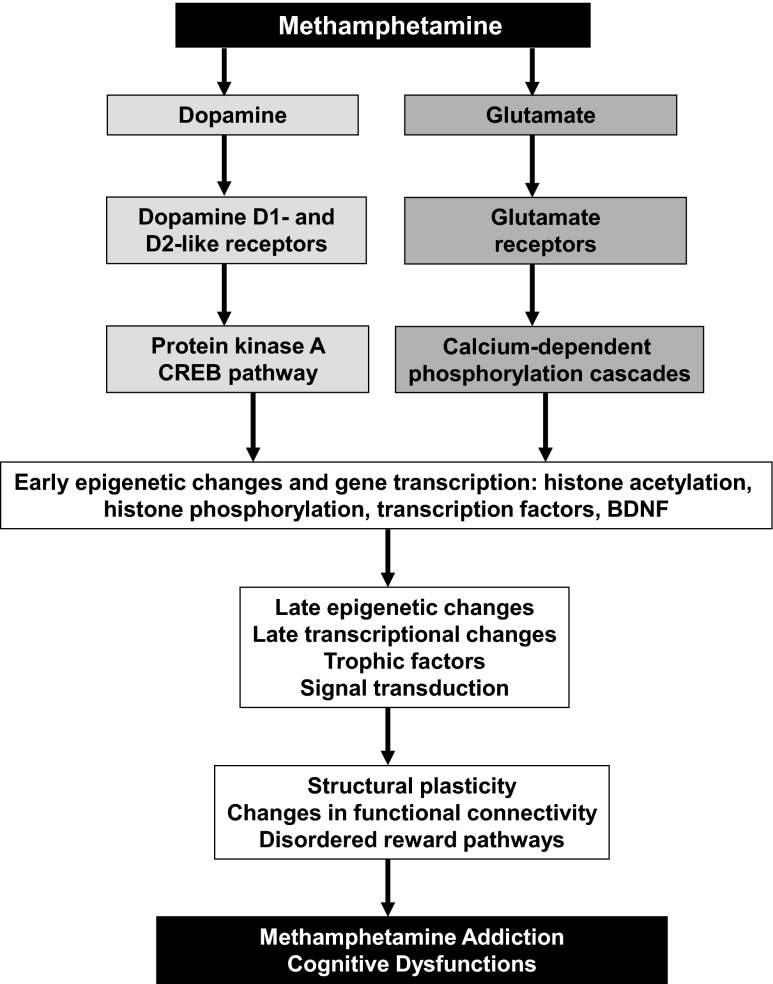
Epigenetic and transcriptional events involved in methamphetamine addiction. This figure describes our theoretical approach to methamphetamine addiction. Although the figure suggests that the biochemical and behavioral effects of methamphetamine appear to involve activation of dopaminergic and glutamatergic pathways, we are cognizant of the fact that other neurotransmitter systems might also participate in causing addiction and associated neuropsychiatric consequences. Activation of these neurotransmitter systems is followed by stimulation and/or inhibition of epigenetic and transcriptional events that generate compulsive abuse of the drug. These compulsive behaviors might also be secondary to a cortical disinhibition-induced subcortical hyperconnection syndrome that is characterized by specific cognitive changes in human methamphetamine addicts

Methamphetamine addiction is a major public health problem that is accompanied by recalcitrant neuropsychiatric and neuropathological complications [1–4]. The neuropsychiatric adverse consequences include subclinical cognitive deficits [5] that can, nevertheless, negatively impact activities of daily living [6, 7]. The clinical course of treatment for methamphetamine use disorders is also accompanied by variable outcomes and rates of recidivism [2, 8, 9] that are also thought to depend on neuroadaptative and/or neuropathological substrates consequent to repeated exposure to the drug [10, 11]. These adaptive changes appear to include, among others, alterations in gene and protein expression [11–14], some of which appear to influence physiological functions at striatal glutamatergic synapses [15]. It is also likely that the behavioral transition from occasional use of psychostimulants to drug addiction may involve a shift of control over drug intake from the ventral to dorsal striatum when the use of drugs becomes truly habitual and compulsive [16]. This transition to addictive behaviors appears to depend, in the case of some drugs, on transcriptional and epigenetic plastic changes in the brain [17, 18]. Similarly, several studies have reported that methamphetamine can significantly influence the expression of many genes in the nucleus accumbens and dorsal striatum after both acute and chronic administration of the drug [14, 19–23]. Although these studies have suggested that administration of methamphetamine might be associated with transcriptional neuroadaptations, much remains to be done in order to further dissect the molecular pathobiology of methamphetamine addiction. In our laboratory, we have envisioned methamphetamine use disorder as a progressive neuropsychiatric disorder that results from a diversity of altered gene expression in the dorsal striatum and other brain regions [10, 11, 15]. In addition, we and others have proposed that these transcriptional changes might be dependent on persistent, yet reversible, epigenetic modifications that drive or inhibit the expression of specific gene networks that regulate cellular and synaptic functions and behavioral responses to the drug [11, 12, 15]. Together, the epigenetically determined changes in gene expression and associated changes in protein levels might then lead to cognitive deficits observed in some methamphetamine-addicted individuals ([10], see Fig. 1). The present review was thus written to provide a summary of our more recent work in transcriptional effects of METH self-administration. The review will also serve to expand on our previous discussion of methamphetamine-induced transcriptional effects in the brain [11].

Towards that end, we will review recent genome-wide transcriptional data collected from the dorsal striatum of rats that had self-administered methamphetamine using a long-access paradigm [24]. We chose the striatum because it is an integral part of a circuit that regulates reward and habit forming [25, 26], both of which are core elements of addiction [10, 27]. We will also describe several gene networks that are affected during both early and late withdrawal times after cessation of methamphetamine self-administration. Moreover, we will touch on the evidence that methamphetamine intake is associated with some epigenetic changes in the dorsal striatum. These results will be discussed within the context of the need to generate novel hypotheses to elucidate the biological substrates of methamphetamine addiction.

## Early Transcriptional and Epigenetic Changes in the Methamphetamine Self-Administration Model

Studies of epigenetic and transcriptional changes associated with drug addiction have focused mostly on the effects of cocaine on gene expression and/or histone modifications in various brain regions [28, 29]. The epigenetic and transcriptional effects of cocaine have been reviewed at great length [17, 18]. However, very few studies have been conducted on the transcriptional and/or epigenetic effects of methamphetamine self-administration. Authors focusing on methamphetamine self-administration have reported on the effects of these behavioral manipulations on dopaminergic [24, 30, 31] and glutamatergic [32] systems as well as c-FOS [13] and brain-derived neurotrophic factor (BDNF) [33] protein expression. Others have reported that methamphetamine self-administration can negatively impact cognitive function [34–36] and cortical electrophysiology [37]. In addition, the effects of withdrawal from extended methamphetamine self-administration were found to be related to the survival of hippocampal progenitor cells [34]. Moreover, withdrawal from extended methamphetamine self-administration was also accompanied by a dysphoric-like state, the neurobiological basis of which is not known [38]. Taken together, the extended methamphetamine self-administration model appears to result in varied clinical and neurobiological outcomes. Nevertheless, there is very little information on the transcriptional effects of similar models of methamphetamine addiction. In an attempt to fill that gap, we have conducted and are continuing to conduct studies to investigate genome-wide transcriptional and epigenetic effects of methamphetamine in the hope of discovering specific substrates for methamphetamine-induced multifaceted behavioral and biochemical effects.

**Fig. 2:**
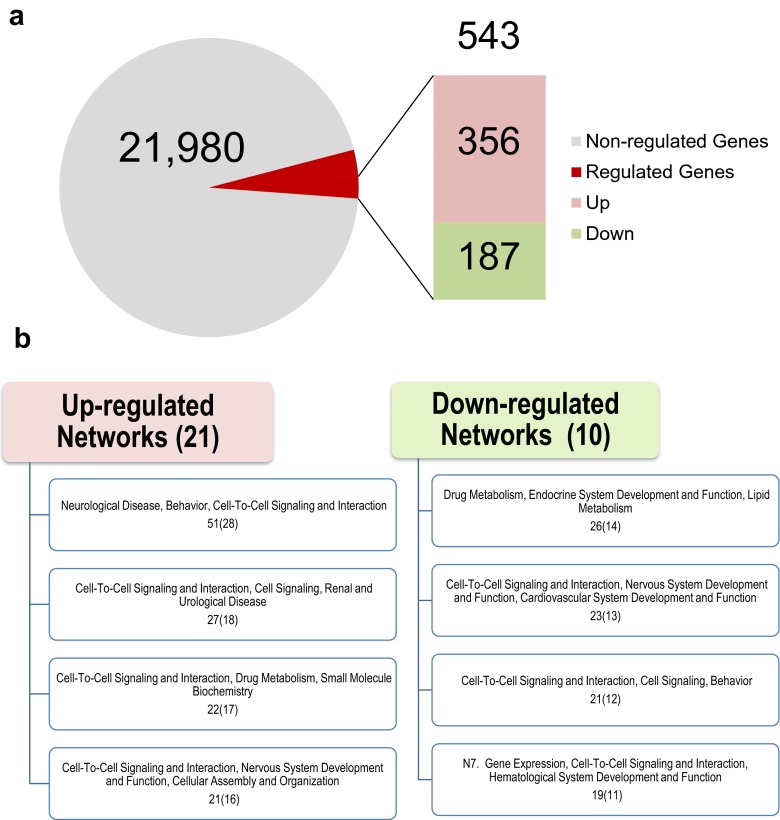
Microarray analysis of gene expression measured in the rat striatum at 2 h after cessation of methamphetamine self-administration. **a** Description of microarray results. The total number of genes (21,980) on the array is shown within the *light grey area* of the circle. Also listed is the total number of genes (543) that are regulated by methamphetamine. The *light pink box* represents the number (356) of upregulated genes whereas the *light green box* shows the number (187) of downregulated genes. **b** Molecular networks of genes differentially affected by methamphetamine self-administration. These networks were generated using Ingenuity Pathway Analysis. The networks are ranked according to their scores, and eight networks of interest are shown. The number of genes in each network is shown in parentheses. Note that several of the networks contain genes that participate in cell-to-cell signaling and interactions

In the experiments being reviewed here, we have used an extended-access model of intravenous methamphetamine self-administration for eight consecutive daily sessions, with the control rats receiving yoked saline injection [11, 24]. The rats were given access to methamphetamine for 15 h per day and were euthanized 2 h after the last session. As described by others [39], rats exposed to extended daily sessions escalate their intake of methamphetamine. More details of the long-access self-administration paradigm can be found in our two recent papers on the subject [11, 24]. Global gene expression was measured in striatal tissues using Illumina 22K Rat microarrays. Detailed experimental protocols for tissue collection, RNA extraction, and performance of microarray analyses can also be found in our many publications on this subject [11, 14] and will not be described at length here. As reported by Krasnova et al. [11], we found that 543 transcripts were differentially expressed using a cutoff value of 1.7-fold (*p* < 0.05) (Fig. 2a). Using similar criteria, we have been able to replicate array expression data from nucleus accumbens, dorsal striatum, or midbrain by using quantitative PCR [14, 19, 40]. For the microarray data described here, Krasnova et al. [11] had also used quantitative PCR to confirm methamphetamine self-administration-induced changes in the expression of several immediate early genes (IEGs), neuropeptides, and plasticity-related genes. Of the methamphetamine-regulated genes, 356 showed increased expression whereas 187 showed decreased expression in the striatum. These genes were analyzed for networks and molecular functions by using Ingenuity Pathways Analysis (Ingenuity Systems). Figure 2b shows that methamphetamine can regulate many biological processes in the dorsal striatum. Specifically, methamphetamine caused upregulation of transcripts that are components of gene networks for neurological disease, cell-to-cell signaling and interaction, nervous system development and function, as well as cellular assembly and organization. Downregulated networks include genes that participate in drug metabolism, endocrine system development and function, cell-to-cell signaling and interaction, and control of gene expression (Fig. 2b). The observation that the drug alters the expression of a large number of transcripts is consistent with the varied clinical manifestations of methamphetamine-addicted patients [4, 5]. These clinical presentations include deficits in executive and memory functions, depression, and psychosis [4, 41]. Our gene expression data thus raise the intriguing possibility that there are subpopulations of methamphetamine addicts who might respond differentially to pharmacological therapeutic approaches.

**Fig. 3:**
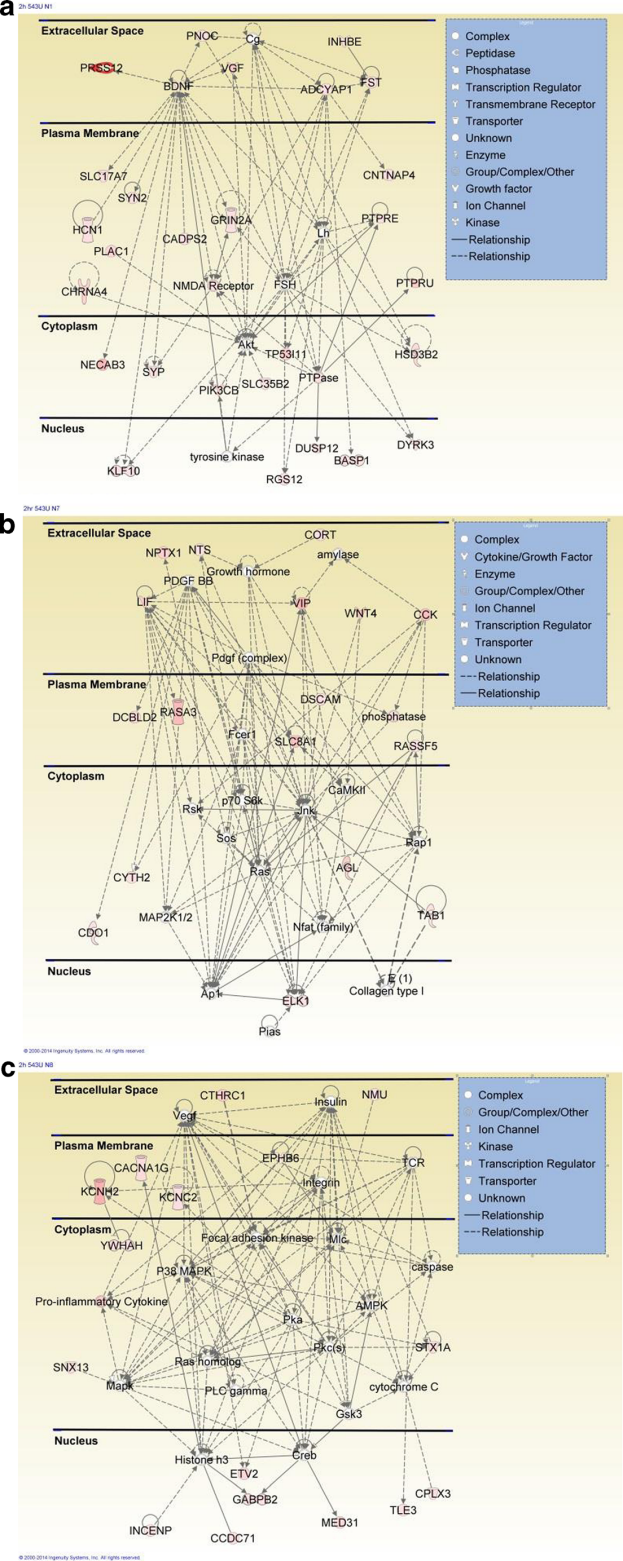
Methamphetamine self-administration causes differential expression of genes involved in several networks. **a** A network of genes involved in neurological disease, behavior, and cell-to-cell signaling and interaction. This list includes BASP1, BDNF, and some phosphatases. **b** A network of genes that participate in cell-to-cell signaling and small molecule metabolism. These genes include CCK, ELK1, and neurotensin. **c** A network of upregulated genes involved in nervous system development and function as well as cellular assembly and organization. Among these genes are neuromedin U and syntaxin 1A. These gene networks emphasize the complex molecular effects of methamphetamine in the brain

Given the multifaceted effects of methamphetamine in the central nervous system (CNS) that include decreased dopamine (DA) and serotonin levels in the dorsal striatum, decreased striatal dopamine transporters, and abnormal glucose metabolism [42–44], it is of interest that several genes that participate in the regulation of transcription, including brain abundant signal protein/brain acid soluble protein 1 (BASP1) (Fig. 3a), ETS domain-containing protein (ELK1) (Fig. 3b), and Kruppel-like zinc finger 10 (KLF10) (Fig. 3a), are upregulated by the drug (Table 1). Interestingly, BASP1 was discovered in rat brain about two decades ago [45]. BASP1 attaches to plasma membrane in nerve terminals [46] and can modify adjoining membrane region through interactions with phosphatidylinositol 4,5-bisphosphate (PIP2) [47]. BASP1 has also been shown to regulate actin cytoskeleton dynamics [48] and to be involved in initiating neurite outgrowth [49]. In addition to its role at nerve terminals, BASP1 was found to be a co-repressor for the Wilms’ tumor suppressor protein (WT1) [50]. BASP1 is found in the nucleus where it is localized on the promoters of WT1 target genes [51, 52]. BASP1 acts by recruiting histone deacetylase 1 (HDAC1) to cause suppression of WT1 target genes [53]. Thus, the identification of these novel effects of methamphetamine suggests that BASP1 might participate in methamphetamine-mediated decreases in striatal gene expression (see Fig. 2b, Table 1). This potential epigenetic effect of methamphetamine is supported by our recent data that identified HDAC1 as an important regulator of methamphetamine-induced changes in the expression of striatal glutamate receptors [15].

**Table 1:** Partial list of 2-h METH-upregulated genes in comparison to 1-month group

Symbol	Entrez gene name	Fold change	
		2 h	1 month
Calcium ion binding			
CADPS2	Ca++-dependent secretion activator 2	2.56	−1.35
NECAB3	N-terminal EF-hand calcium-binding protein 3	8.53	−1.94
Cell adhesion			
CNTNAP4	Contactin-associated protein-like 4	3.71	−1.03
DSCAM	Down syndrome cell adhesion molecule	1.78	−1.49
Cell growth			
DCBLD2	Discoidin, CUB and LCCL domain containing 2	4.63	−1.47
INHBE	Inhibin, beta E	3.44	1.25
Cell migration			
SNX13	Sorting nexin 13	2.12	1.34
Development			
PLAC1	Placenta-specific 1	5.11	−1.70
DNA binding			
ETV2	Ets variant 2	4.73	1.00
KLF10	Kruppel-like factor 10	1.97	1.25
TP53I11	Tumor protein p53 inducible protein 11	6.34	−1.26
Ion transport			
CACNA1G	Calcium channel, voltage-dependent, T type, alpha 1G subunit	4.41	−1.36
HCN1	Hyperpolarization-activated cyclic nucleotide-gated K^+^ channel 1	2.92	−1.99
KCNC2	Potassium voltage-gated channel, Shaw-related subfamily, member 2	1.90	−2.56
KCNH2	Potassium voltage-gated channel, subfamily H, member 2	9.90	−1.77
SLC17A7	Solute carrier family 17, member 7	3.95	−1.06
SLC35B2	Solute carrier family 35, member B2	1.92	−1.39
SLC8A1	Solute carrier family 8 (sodium/calcium exchanger), member 1	7.56	1.04
Metabolism			
AGL	Amylo-alpha-1, 6-glucosidase, 4-alpha-glucanotransferase	6.39	−2.02
CDO1	Cysteine dioxygenase type 1	1.76	−1.56
HSD3B2	hydroxy-δ-5-steroid dehydrogenase, 3 β- and steroid δ-isomerase 2	4.52	−1.23
Neuropeptide/hormone activity			
CCK	Cholecystokinin	7.93	−1.47
CORT	Cortistatin	3.53	1.30
FST	Follistatin	2.20	1.00
NMU	Neuromedin U	3.84	−1.00
NTS	Neurotensin	3.08	−1.86
PNOC	Prepronociceptin	3.42	−2.35
VIP	Vasoactive intestinal peptide	8.89	−5.03
Neurotransmitter transporter			
CPLX3	Complexin 3	3.51	1.93
Neurotransmitter release			
STX1A	Syntaxin 1A (brain)	2.30	−1.10
SYN2	Synapsin II	2.12	1.03
SYP	Synaptophysin	1.74	−1.53
Protein binding			
INCENP	Inner centromere protein antigens 135/155 kDa	2.33	1.16
MED31	Mediator complex subunit 31	4.78	1.19
Protein transport			
CYTH2	Cytohesin 2	1.75	−1.40
Proteolysis			
PRSS12	Protease, serine, 12 (neurotrypsin, motopsin)	36.14	1.40
Signal transduction			
ADCYAP1	Adenylate cyclase activating polypeptide 1 (pituitary)	2.32	−1.15
CHRNA4	Cholinergic receptor, nicotinic, alpha 4 (neuronal)	5.60	−1.05
CTHRC1	Collagen triple helix repeat containing 1	6.12	−1.80
DUSP12	Dual-specificity phosphatase 12	2.29	−1.73
DYRK3	Dual-specificity tyrosine-(Y)-phosphorylation regulated kinase 3	4.62	1.45
EPHB6	EPH receptor B6	1.99	−1.47
GRIN2A	Glutamate receptor, ionotropic, *N*-methyl d-aspartate 2A	1.84	−1.32
NPTX1	Neuronal pentraxin I	5.79	1.06
PIK3CB	Phosphatidylinositol-4,5-bisphosphate 3-kinase, catalytic subunit β	5.50	−1.33
PTPRE	Protein tyrosine phosphatase, receptor type, E	3.18	1.14
PTPRU	protein tyrosine phosphatase, receptor type, U	6.55	−1.31
RASA3	RAS p21 protein activator 3	8.44	−1.20
RASSF5	Ras association (RalGDS/AF-6) domain family member 5	1.70	−1.68
RGS12	Regulator of G-protein signaling 12	2.14	−1.17
TAB1	TGF-beta activated kinase 1/MAP3K7 binding protein 1	3.94	−1.02
WNT4	Wingless-type MMTV integration site family, member 4	7.48	−1.69
YWHAH	Tyrosine 3-monooxygenase/tryptophan 5-monooxygenase activation protein	1.83	−1.58
Transcription			
BASP1	Brain abundant, membrane attached signal protein 1	1.74	−1.40
CCDC71	Coiled-coil domain containing 71	2.96	−1.60
ELK1	ELK1, member of ETS oncogene family	3.72	−1.23
GABPB2	GA binding protein transcription factor, beta subunit 2	3.48	−2.07
LIF	Leukemia inhibitory factor	6.10	−1.97
TLE3	Transducin-like enhancer of split 3	1.74	−1.43
Trophic factor			
BDNF	Brain-derived neurotrophic factor	3.28	−1.02
VGF	Nerve growth factor inducible	7.02	−1.58

The experimental model and microarray analyses were performed as described in the text. This partial list of genes was generated from the 2 h microarray data. The expression data were then compared to the fold changes in expression obtained for these genes after 1 month of withdrawal. To be included, the genes had to meet the inclusion criteria: + 1.7-fold at *p* < 0.05 at the 2 h time point

Another gene of interest whose expression is upregulated in this model is Elk1 (Fig. 3b, Table 1) which is a member of a ternary complex factor (TCF) subgroup of the family of the E-twenty-six (ETS)-domain transcription factors [54]. Elk1 is an important target of the canonical extracellular signal-regulated kinases 1 (ERK1) and 2 (ERK2) pathways [55, 56]. In the general context of addiction, various pharmacological agents have been shown to activate ERK1 and ERK2 in a DA and glutamate-dependent manner [57–60]. ERK1 and ERK2 are two very closely related kinases whose activation is dependent on their phosphorylation by mitogen-activated protein kinases [61, 62]. ERKs, in turn, phosphorylate ELK1 [55, 56]. ELK1 is widely distributed in the adult rat brain [63] and is involved in the regulation of functionally distinct networks of genes [64], including c-fos [65, 66] and early growth factor 1 (Egr1) in the striatum [63, 67]. Thus, the methamphetamine-induced expression of ELK1 suggests that the drug might have altered the expression of some genes, in part, by activating the MAP-ERK-ELK1 pathway. This suggestion is consistent with previous demonstration that some amphetamine analogs can increase ERK phosphorylation [68–70] and with the report that ELK1 activation is involved in cocaine-induced behavioral and molecular alterations [71]. This notion is also supported by the fact that the ERK mitogen-activated protein (MAP) kinase pathway is involved in cognitive processes [72] that are involved in the development of addiction [10].

Further evidence for the involvement of phosphorylation/dephosphorylation cascades in methamphetamine addiction is also provided by the observation of methamphetamine-induced increased phosphorylation of cyclic AMP response element binding (CREB) protein in the rat striatum [11]. CREB is a member of the CREB/activating transcription factor (ATF) family of transcription factors and is phosphorylated by cAMP-dependent protein kinase A (PKA) and other kinases [73]. Interestingly, the MAPK/ERK cascade has been shown to phosphorylate both ELK1 and CREB to increase c-fos and Egr1 expression in the striatum [67] and to control long-term potentiation-dependent transcription in the hippocampus [74]. CREB phosphorylation is indeed involved in the propagation of signals from various neurotransmitters [75–77]. CREB phosphorylation also promotes the recruitment of co-activators, such as CREB-binding protein (CBP)/p300, to the basal transcriptional machinery, a process that is followed by increased expression of CREB target genes [78]. These genes include immediate early genes (IEGs) such as arc, c-fos, egr1, several dual-specificity phosphatases (DUSPs), as well as BDNF [79, 80]. Consistent with these observations, we found that methamphetamine self-administration was accompanied by increased c-fos and BDNF at the early time point of withdrawal from drug taking by the rats [11]. These results are consistent with the report of Cornish et al.[13] who had reported significant increases in c-Fos protein expression in the dorsal striatum and cortex after a 3-week period of METH self-administration of 2-h daily sessions. Their paradigm is different from the one used in our study because the rats had 15-h access to drug for 8 days [11]. In both models, nevertheless, the METH effects might have occurred via stimulation of striatal DA receptors, followed by activation of various kinases, phosphorylation of CREB, and consequent CREB-mediated transcription [81–84]. This idea is supported by our findings that METH self-administration was accompanied by increased recruitment of phosphorylated CREB on the promoters of *c-fos*, *fosB*, and *Bdnf* [11]. In addition, these observations indicate that *c-fos*, *fosB*, and *Bdnf* genes might be co-regulated in some brain regions at both epigenetic and transcriptional levels and may work together to maintain some of the plasticity changes that might generate the regional substrates of methamphetamine addiction. This conclusion is supported by the demonstration of the important roles that activation of CREB and IEGs, including c-fos and egr1, plays in processes related to learning and memory formation [85–87]. The methamphetamine-induced increases in *Bdnf* messenger (m)RNA expression are accompanied by increased BDNF protein expression at the early time point. Our observations of METH self-administration-induced BDNF expression is consistent with those of McFadden et al. [33] who also reported that METH self-administration was accompanied by increased BDNF expression in the rat hippocampus. Taken together, it appears that METH self-administration might influence the expression of certain genes in various brain regions including the cortex, striatum, and hippocampus [11, 13, 33]. These results are also consistent with clinical studies that had reported increases in BDNF levels in the plasma of chronic METH users [88]. Moreover, this notion is supported by the possibility that BDNF signaling may play an integral part in producing plastic changes that lead to addiction [89] through processes that involved changes in the expression of proteins such as synapsin and synaptophysin that are involved in synaptic functions [90, 91]. Our findings that methamphetamine does increase the expression of synaptophysin (Fig. 3a), syntaxin 1A (Fig. 3c), and synapsins [11] provide further evidence that altered synaptic plasticity is at the core of methamphetamine self-administration. Synapsins are a family of phosphoproteins that are located in presynaptic terminals [92, 93]. They promote synaptogenesis and regulate vesicle dynamics and neurotransmitter release [94–96], functions that are dependent on phosphorylation/dephosphorylation events [97, 98]. Thus, our observations of methamphetamine-induced changes in the expression of these synaptic proteins might be relevant to the report that repeated methamphetamine exposure causes changes in the density of dendritic spines on medium spiny neurons [99], changes that are dependent on activation of the BDNF-tyrosine kinase receptor, type 2 (TrkB) signaling pathway [100].

**Fig. 4:**
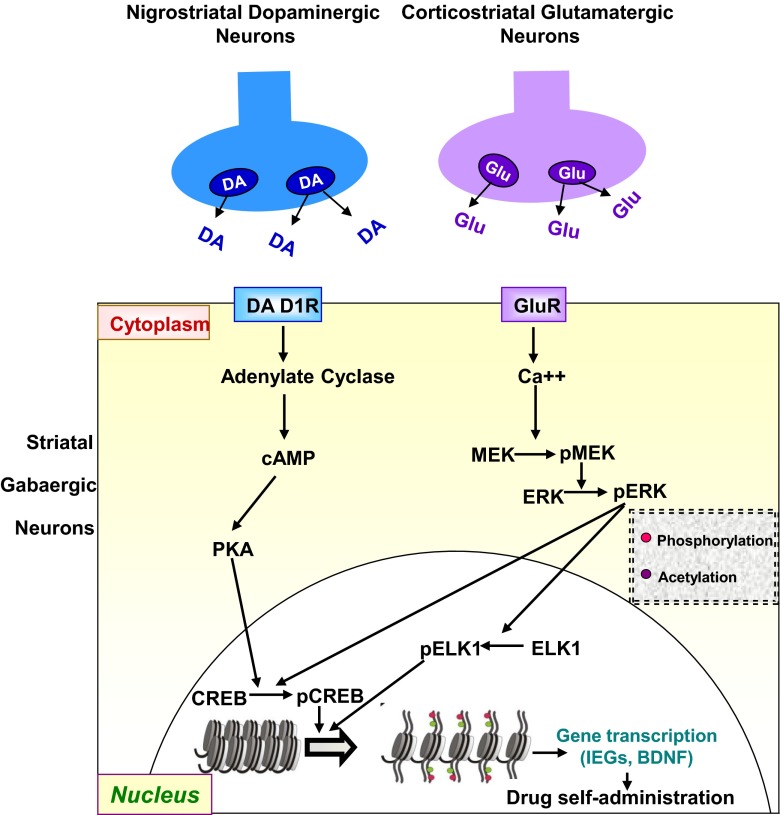
Methamphetamine self-administration causes co-activation of CREB- and ELK1-dependent pathways in the rat striatum. The scheme shows the potential activation of the MAPK-ERK-ELK1 and PKA-CREB pathways via stimulation of both dopamine and glutamate receptors. The theoretical scheme also suggests that activation of these two pathways would also lead to chromatin changes that might be responsible for the changes in the expression of genes such as BDNF and some immediate early genes (*IEGs*). Although the scheme has focused on the dopaminergic and glutamatergic systems for the sake of simplicity, other neurotransmitter systems including neuropeptides might also participate in the long-term alterations in gene expression in the striatum (see Krasnova et al.[11])

Related to the discussion of the role of a potential convergence of the MAP/ERK/ELK1 and CREB phosphorylation pathways in methamphetamine addiction (Fig. 4), it is of interest that the microarray analysis also identified several phosphatases, including dual-specificity phosphatase 12 (DUSP12), protein tyrosine phosphatase receptor, type E (PTPRE), and protein tyrosine phosphatase receptor, type U (PTPRU) that were also upregulated by methamphetamine self-administration (Fig. 3a, Table 1). Protein phosphorylation/dephosphorylation processes are major mechanisms that regulate signal transduction pathways [101]. These processes are tightly regulated by protein tyrosine kinases (PTKs) and phosphatases (PTPs) that are highly expressed in the brain [101]. Other members of the general PTP family can also remove phosphate groups from phosphoserine, phosphothreonine, and phosphotyrosine residues and constitute a family of versatile enzymes called DUSPs [102]. PTPs are also divided into receptor-like or membrane-bound PTP (RPTP) and non-receptor or cytosolic, soluble PTPs [103]. DUSPs serve to provide negative feedback to MAPK and cyclin-dependent kinase (CDK) pathways by deactivating these enzymes via dephosphorylation events [102]. Because of their ubiquity, the DUSPs are involved in the regulation of many cellular functions [104]. However, in contrast to other DUSPs such as DUSP1-DUSP10, the role of DUSP12, an atypical DUSP [105], in the central nervous system has not been investigated actively. Nevertheless, DUSP12 has been shown to interact with Hsp70, and its overexpression protects against heat shock- and hydrogen peroxide-induced cell death, a function that requires its phosphatase activity [106]. Its antioxidative properties might be due to the fact that DUSP12 can sense oxidative stress by its thiol-rich zinc-coordinating domain [107]. Although the role of DUSP12 in methamphetamine addiction remains to be clarified, its increased expression in the present model is consistent with the fact that acute injections of the drug can cause oxidative stress in various brain regions [3, 108]. Increased markers of striatal toxicity have also been found in rats that self-administered methamphetamine [24] in a pattern similar to the one used in the present report. Together, these observations suggest that methamphetamine self-administration may result in oxidative stress in the rat striatum.

In addition to DUSP12, PTPRE and PTPRU were also upregulated in the methamphetamine self-administration model. PTPRE and PTPRU are members of the receptor-like PTPs [109] that are expressed in the brain [110–113]. PTPRE has been shown to regulate voltage-gated potassium channels in Schwann cells [114]. Of interest, we observed significant increases in the expression of KCNC2 and KCNH2 in the methamphetamine-treated rats (Fig. 3c, Table 1). Related to this discussion is the fact that PTPRE can inhibit ERK1 and ERK2 kinase activities and block ELK1-induced transcriptional activity [115] in a fashion similar to the DUSPs [102]. PTPRU (also called RPTP lambda or psi) is co-localized with cell adhesion molecules including catenin and E-cadherin [116]. The phosphatase contains a large region that is homologous to the intracellular cellular domain of cadherins and interacts directly with and dephosphorylates beta-catenin [117], an important component of Wnt signaling [118]. This action of PTPRU leads to inhibition of beta-catenin signaling [119, 120]. PTPRU also participates in Delta/Notch signaling [121]. This phosphatase is highly expressed in the midbrain/hindbrain boundary [122] and plays important role in the development of the midbrain [120]. Interestingly, PTPRU mRNA expression is regulated by the combined action of Nr4a2 and Pitx3 [111], both of which are upregulated by methamphetamine administration [14, 21]. These observations support the view that methamphetamine self-administration can activate gene networks that participate in various brain regulatory functions. Our results also suggest that the drug might cause activation of phosphorylation/dephosphorylation cascades to regulate and balance the activity of multiple signaling pathways during the transition to escalating methamphetamine intake in this model (see Fig. 4 for a scheme). Our results also support the thesis that drug addiction is related to changes in synaptic plasticity that may be mediated by the activation of a combination of molecular networks that impact neurotransmission in the dorsal striatum. Finally, the idea that protein phosphatases might be involved in addiction is supported by the observation that striatal PTP alpha promotes alcohol addiction in rodents [123].

Methamphetamine self-administration is also accompanied with increases in KLF10 expression (Fig. 3a). KLF10 is a member of the family of Sp1/Kruppel-like zinc finger transcription factors [124, 125]. KFL10 contains three repression (R1–R3) domains at the N-terminal [126], with the R1 domain being important for its interaction with the co-repressor, Sin3A, which suppresses gene expression by recruiting HDACs [127]. KLF10 can also suppress transcription via its interaction with Jumonji AT-rich interactive domain 1B/lysine-specific demethylase 5B (JARID1B/KDM5B) [128], an enzyme that removes methyl residues from trimethylated lysine 4 of histone 3 (H3K4me3) [129], a marker that is associated with active gene transcription [130]. The increased KLF10 expression might therefore be an attempt to correct methamphetamine-induced increased H3K4me3 abundance in the striatum [11]. The potential increased expression of repressor proteins during methamphetamine self-administration is consistent with the observations of decreased expression of several gene networks (Fig. 2b) in this model of methamphetamine addiction. This discussion suggests the possibility that KLF10 might be an important regulator of methamphetamine-induced epigenetic events. The potential role for these epigenetic marks in the long-term effects of the drug can also be inferred from the observed downregulation of several gene networks at a later time point of withdrawal from methamphetamine self-administration (see discussion below). In any case, more studies are needed to dissect the role of methylation processes in methamphetamine addiction [12], given the important of this histone mark in various biological functions [131].

## Delayed Transcriptional Changes After Methamphetamine Self-Administration

Methamphetamine-addicted individuals show differential outcomes during the course of various therapeutic modalities [2, 8]. Interviews at 2–3 years after treatment showed that 50 % had returned to using drugs, with 36 % doing so within the first 6 months after the treatment period [8]. Methamphetamine addicts appear to relapse for a multitude of reasons that include pleasure seeking, impulsivity, habits, and pain avoidance [132]. In animal models of methamphetamine addiction, the number of lever pressing for an absent methamphetamine award is higher at later withdrawal times than that observed during early withdrawal [133], a phenomenon that has been referred to as incubation of drug craving [134]. Recently, it was reported that animals that were rendered abstinent from methamphetamine self-administration by response-contingent foot-shocks also demonstrated incubation of methamphetamine craving [135]. These clinical and preclinical results suggest that different molecular changes that occur during early and/or late withdrawal states might differentially influence striatal functions and cause different motoric behavioral outcomes that might manifest as larger number of lever presses at longer withdrawal times [133, 135]. The notion that striatal gene expression changes might play a role in behaviors observed after several weeks of withdrawal is consistent with data from microarray analyses that we describe below.

**Fig. 5:**
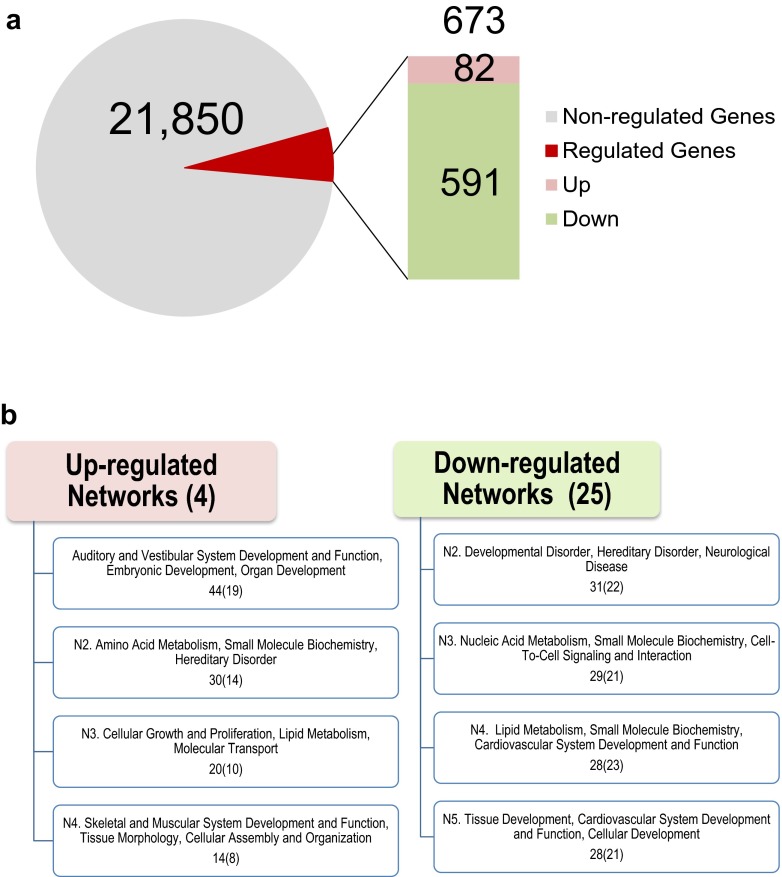
Microarray analysis of striatal gene expression at 1 month after cessation of methamphetamine self-administration. **a** Description of microarray results. The total number of genes (21, 850) measured on these arrays is shown within the *light grey area* of the circle. The number of genes (673) that are regulated by methamphetamine is also shown. The *light pink box* represents the number (82) of upregulated genes whereas the *light green box* shows the number (591) of downregulated genes. **b** Molecular networks of genes differentially affected by methamphetamine self-administration. These networks were generated using IPA. The networks are ranked according to their scores, and eight networks of interest are shown. The number of genes in each network is shown in parentheses. Importantly, very different gene networks are affected at that time point, suggesting considerable differences between early and delayed neuroadaptations after cessation of drug self-administration

In the set of experiments examining the delayed effects of methamphetamine withdrawal, rats underwent the same self-administration procedure described elsewhere [11, 24] and were euthanized at 1 month after the last session. Global striatal gene expression was again measured using Illumina 22K Rat microarrays. We found that 673 transcripts were differentially expressed at that time point (Fig. 5a). Of these methamphetamine-regulated genes, only 82 were upregulated whereas 591 were downregulated. These results are different from those obtained at the early withdrawal time point when the majority of genes were upregulated (see Fig. 2a). The observation of large number of downregulated genes after methamphetamine withdrawal is consistent with previous results showing that methamphetamine can cause increased expression of histone deacetylases (HDACs) in the nucleus accumbens [14] and the dorsal striatum [15]. HDACs are enzymes that can cause histone deacetylation and repression of gene expression [136, 137]. HDACs are important regulators of synaptic formation, synaptic plasticity, and long-term memory formation [138–141]. Several HDACs also appear to play significant roles in various models of drug abuse and addiction [142–147].

The differentially expressed genes were analyzed for networks and molecular functions by using Ingenuity Pathways Analysis (Ingenuity Systems). Figure 5b shows that methamphetamine can regulate many biological processes in the dorsal striatum. Specifically, withdrawal from methamphetamine self-administration is accompanied with upregulation of transcripts that are components of gene networks involved in embryonic and organ development, amino acid metabolism, cellular growth and proliferation, and cellular assembly and organization, among others (Table 2). Downregulated networks include genes that participate in developmental disorders, neurological diseases, cell-to-cell signaling, and cardiovascular development and function (Fig. 5b, Table 2).

**Table 2:** Partial list of 1-month METH-regulated genes in comparison to 2-h group

Symbol	Entrez gene name	Fold change	
		2 h	1 month
Autophagy			
TBC1D14	TBC1 domain family, member 14	1.41	−3.67
Cell cycle			
CCNA1	Cyclin A1	1.57	−1.81
CD82	CD82 molecule	1.05	−1.71
CDC25A	Cell division cycle 25A	1.27	−4.25
CDK4	Cyclin-dependent kinase 4	1.17	−1.7
CHEK2	Checkpoint kinase 2	1.12	−3.59
GADD45G	Growth arrest and DNA-damage-inducible, gamma	1.21	−1.73
Cell differentiation			
BAMBI	BMP and activin membrane-bound inhibitor	1.42	−1.96
DHH	Desert hedgehog	−1.85	3.06
DLX1	Distal-less homeobox 1	1.16	−1.71
LIMD1	LIM domains containing 1	1.72	−2.19
NNAT	Neuronatin	−1.03	−2.11
VPS52	Vacuolar protein sorting 52 homolog	1.26	−2.70
Chromatin remodeling			
ARID2	AT-rich interactive domain 2	1.46	−2.84
ARID4A	AT-rich interactive domain 4A	−1.20	−1.73
CTR9	CTR9, Paf1/RNA polymerase II complex component	1.32	−1.74
EPC1	Enhancer of polycomb homolog 1	1.44	4.78
RNF187	Ring finger protein 187	1.10	−3.91
RNF113A	Ring finger protein 113A	1.22	−1.71
Sp2	Sp2 transcription factor	−1.22	3.14
Coagulation			
PLG	Plasminogen	1.48	−1.94
Cytoskeleton			
KIF4A	Kinesin family member 4A	1.42	−1.95
MFAP1	Microfibrillar-associated protein 1	2.34	−2.17
DNA repair			
MPG	*N*-methylpurine-DNA glycosylase	2.00	−1.88
RAD51	RAD51 recombinase	1.82	−2.03
DNA replication			
POLD1	Polymerase (DNA directed), delta 1, catalytic subunit	−1.02	−1.71
POLH	Polymerase (DNA directed), eta	1.22	−5.38
Growth factor			
HGF	Hepatocyte growth factor	−1.98	−1.87
OSM	oncostatin M	−1.00	−3.9
Homeostasis			
OCM	Oncomodulin	−2.05	2.63
Immune system			
Klra4	Killer cell lectin-like receptor, subfamily A, member 4	−1.93	−3.10
Ion transport			
SLC22A7	Solute carrier family 22, member 7	1.08	−1.85
Metabolism			
PLD4	Phospholipase D family, member 4	1.50	−1.76
PROCA1	Protein interacting with cyclin A1	1.37	−3.54
ALDOB	Aldolase B, fructose-bisphosphate	−1.00	−1.93
Hddc3	HD domain containing 3	1.51	−1.87
Photoreceptor			
RHO	Rhodopsin	−1.22	−3.70
Protein binding			
ANKRD50	Ankyrin repeat domain 50	3.69	4.48
LRRC59	Leucine-rich repeat containing 59	1.15	−1.76
Proteolysis			
MMP13	Matrix metallopeptidase 13 (collagenase 3)	−1.00	−4.18
Signal transduction			
DUSP10	Dual-specificity phosphatase 10	1.43	−3.00
DUSP19	Dual-specificity phosphatase 19	−1.45	−3.41
HIPK3	Homeodomain-interacting protein kinase 3	1.04	1.82
Structural			
LAMB3	Laminin, beta 3	1.12	−2.14
Transcription			
IKZF2	IKAROS family zinc finger 2 (Helios)	1.11	−2.32
JUND	jun D proto-oncogene	−1.14	−1.72
KLF12	Kruppel-like factor 12	2.17	−3.15
LEO1	Leo1, Paf1/RNA polymerase II complex component	1.00	−1.78
LMO1	LIM domain only 1 (rhombotin 1)	−1.34	−1.80
LRCH4	Leucine-rich repeats and calponin homology domain containing 4	−1.52	−2.50
NFYB	Nuclear transcription factor Y, beta	1.38	−2.37
NKX2-4	NK2 homeobox 4	1.29	3.55
RCOR2	REST co-repressor 2	1.19	−4.88
TAL2	T-cell acute lymphocytic leukemia 2	−1.54	−2.08
YY1	YY1 transcription factor	1.35	−2.52
Translation			
EIF2A	Eukaryotic translation initiation factor 2A, 65 kDa	−2.68	3.86
EIF2D	Eukaryotic translation initiation factor 2D	1.37	−1.81

The experimental model and microarray analyses were performed as described in the text. This partial list of genes was generated from the 1 month withdrawal dataset. The gene expression data were then compared to the fold changes obtained for these genes at the 2h time point. To be included, the genes had to meet the inclusion criteria: + 1.7-fold at *p* < 0.05 at the 1 month withdrawal time point

**Fig. 6:**
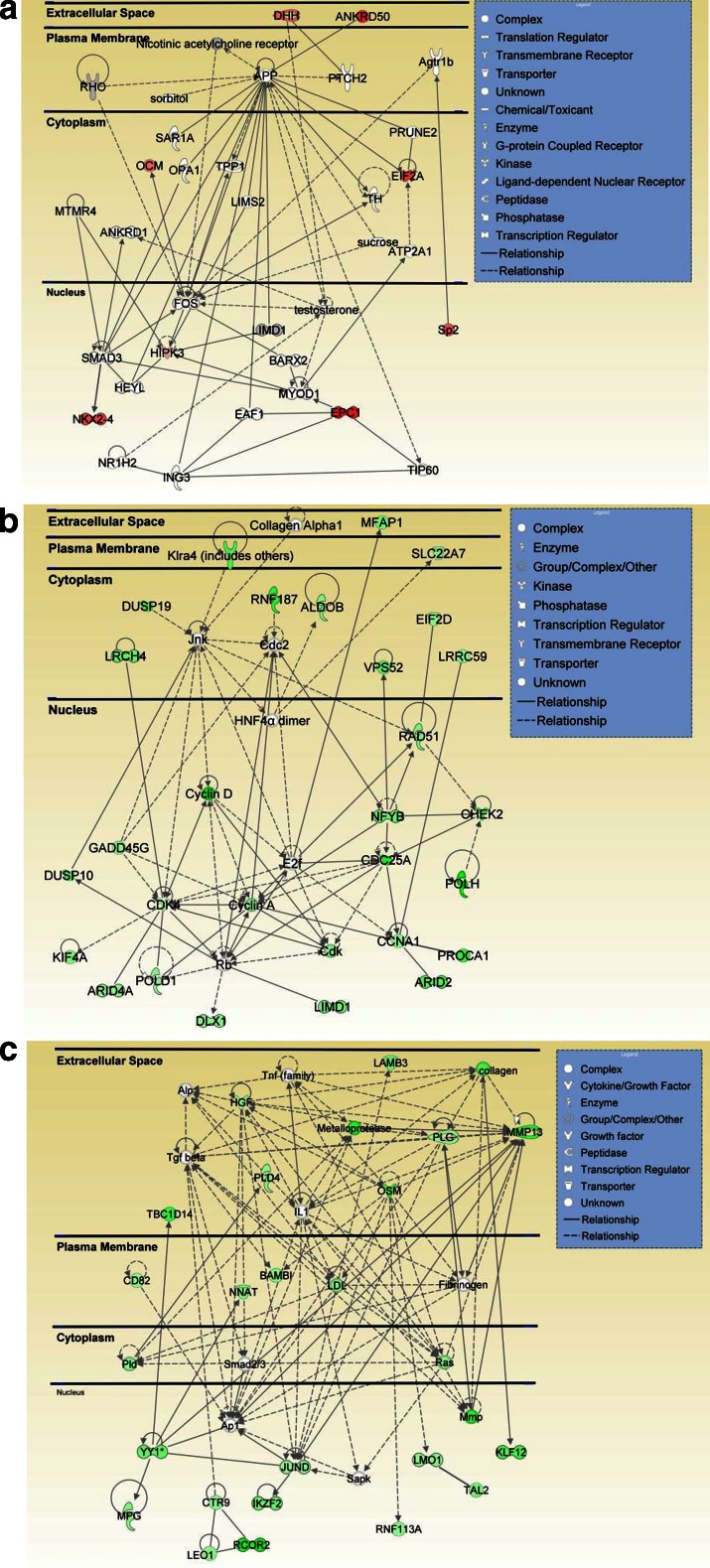
Withdrawal form methamphetamine self-administration causes differential changes in the expression of genes involved in several networks. **a** A network of upregulated genes involved in tissue morphology and cellular assembly. **b** A network of downregulated genes that participate in cell cycle, DNA replication, and repair, as well as cell death and survival. **c** A network of downregulated genes involved in cellular and tissue development. This network includes several transcription regulators including JunD, KLF12, and RCOR2

One of the upregulated genes of interest is the eukaryotic initiation factor alpha (eIF2alpha) (Fig. 6a, Table 2) because of its potential involvement in memory formation [148]. Methamphetamine-addicted individuals are known to suffer from memory deficits that may remain obvious even after long periods of drug withdrawal [44]. The clinical observations suggest that methamphetamine addiction might be associated with abnormalities in protein synthesis since long-term memory is dependent on de novo protein synthesis that is regulated by eIF2alpha [148, 149]. Newly translated proteins are thought to indeed contribute to the formation of new synapses that are involved in long-term storage of memory traces [150, 151]. In eukaryotes, translation initiation is stimulated by the delivery of initiator methionyl-tRNA in the form of an eIF2*GTP*Met-tRNA ternary complex [152]. This complex also includes eIF1A and eIF3 and binds near the 5′ end of mRNAs to initiate translation [153]. Thus, the methamphetamine-induced increased eIF2alpha mRNA suggests the possibility that there might be increased expression of certain proteins at this delayed time point after withdrawal from the drug. However, the possibility also exists that these changes might constitute compensatory increases due to decreased expression of a large number of proteins, given our observations that many transcripts are downregulated in the methamphetamine-treated rats (Table 2). This idea is also consistent with our demonstration that rats that had undergone the methamphetamine self-administration paradigm showed decreased BDNF, TrkB, and delta fosB protein levels at the 1-month withdrawal time point ([11]; see discussion above). A recent study has also reported that there is a fine regulation of transcription and translation to modulate gene expression under different stressful conditions including oxidative stress and heat shock [154]. It may be therefore possible to conclude that these biochemical events might trigger compensatory responses that included increased eIF2alpha transcription because exposure to methamphetamine causes oxidative stress, heat shock, and endoplasmic reticulum (ER) stress [108, 155, 156].

It is also of interest to discuss the changes in eIF2alpha in relationship to the cognitive deficits observed in some methamphetamine abusers [44]. For example, another neuropsychiatric disorder in which patients show cognitive deficits is Alzheimer’s disease (AD) [157]. The brains of these patients show accumulation of beta-amyloid [158]. AD brains also show increased levels of activated and phosphorylated double-stranded RNA-dependent kinase (PKR) [159]. Animal models of AD also show activated PKR [159, 160]. PKR is a serine-threonine protein kinase that is involved in cellular responses to oxidative stress, ER stress, and decreased expression of trophic factors [161]. Importantly, PKR phosphorylates eIFalpha and leads to decreased protein synthesis [152, 162]. Another eIF2alpha kinase, the ER-responsive PKR-like ER-resident kinase (PERK) [163], is also activated in animal models of AD [164]. Together, these observations had suggested that these stress-responsive kinases might play an important role in the cognitive manifestations of AD. This idea was tested by Ma et al. [165] who reported that PERK deletion prevented deficits in protein synthesis and in spatial memory in mice models of AD. These findings are relevant to our discussion of methamphetamine addiction because methamphetamine also activates the ER PERK-dependent pathway [156]. Therefore, the possibility exists that cognitive deficits observed in methamphetamine-addicted individuals might also be due to ER stress-dependent PERK-mediated eIF2alpha phosphorylation, followed by decreased expression of plasticity-related proteins as demonstrated for BDNF and TrkB protein expression in this methamphetamine self-administration model [11]. The idea that there might be a general reduction of protein synthesis in the methamphetamine self-administration model is supported by the observation of decreased expression of another translation initiation factor, eIF2D [166] (Fig. 6b).

In addition to the possible involvement of changes in protein synthesis in the manifestation of methamphetamine addiction, our study has documented substantial decreases in the levels of transcripts that are transcription regulators (Fig. 6b–c, Table 2). These include AT-rich interactive domain 2 (ARID2) (Fig. 6a), ARID4A (Fig. 6b), junD (Fig. 6c), and leucine-rich repeats and calponin homology (CH) domain containing 4 (LRCH4) (Fig. 6b), among others (Table 2). JunD is an intronless gene [167] that is regulated at the translational level [168]. JunD is a member of the activating protein 1 (AP1) family of transcription regulators [169, 170]. The AP1 complexes contain members of the FOS (c-fos, fosB, Fra1, Fra2), JUN (c-jun, junB, and junD), and ATF/CREB (multiple ATFs) families [171, 172]. The Jun family members can homodimerize or heterodimerize with FOS family members to regulate gene expression. AP1 complexes also differ in their binding and transactivating efficiencies based on their compositions and they can either activate or repress the transcription of genes that mediate multiple cellular functions [171, 173, 174]. JunD binds to the TPA-responsive element when it is in the form of homodimers or heterodimers with FOS and JUN family members [175]. In contrast, it binds CRE when it is in the form of heterodimers with ATF family members [176, 177]. The JUN family members also display different patterns of expression during cell cycle progression, with JunD showing no significant changes [178]. JunD protects against p53-induced cell death [179] and regulates the expression of genes involved in cellular antioxidant responses [180, 181] and inflammatory responses [182, 183]. JunD is also involved in nerve growth factor (NGF)-induced upregulation of Nr4a1 in PC12 cells [184]. JunD also dimerizes with Fra2 to mediate NGF-mediated changes in gene expression in PC12 cells [185]. The protein also dimerizes with FosB to regulate okadaic acid-induced transcriptional changes [186] and glutamate-mediated death [187]. JunD also regulates the expression of proenkephalin expression in in vitro models [188]. Altogether, these studies had identified a larger number of JunD target genes in various organ systems (see [183] for an extensive list of JunD-regulated genes). JunD is also highly expressed in the nervous system [189–191] where its expression is responsive to methamphetamine administration [22]. The observations of decreased JunD expression after 1 month of withdrawal from methamphetamine self-administration are consistent with our previous observations that repeated methamphetamine injections for 2 weeks caused decreases in striatal JunD expression [22]. The decreased JunD expression suggests that alterations in JunD expression might play an important role in regulating the expression of the large number of genes that are downregulated at the 30-day withdrawal time point. Because one of JunD binding partners, deltaFosB, is also downregulated at that time [11] and because deltaFosB is also a key regulator in gene expression in other models of drug addiction [17], our findings suggest that, together, the downregulation of both JunD and deltaFosB model might serve to generate the increased motoric behaviors (e.g., increased lever presses) observed after lengthy withdrawals from methamphetamine self-administration [135]. Together, these observations implicate AP1 transcription factors as important players in addiction processes.

Another transcription regulator of interest is AT-rich interactive domain 2 (ARID2) (Fig 6b). ARID2 [192, 193] is a subunit of the polybromo- and BRG1-associated factor (PBAF) chromatin-remodeling complex that regulates gene expression [194, 195]. The protein contains an N-terminal AT-rich DNA binding domain and two C-terminal motifs that serve to bind DNA [196]. The ARID gene family consists of 15 members that are conserved from yeast to humans [197]. The ARID2-containing complex uses energy generated by ATP hydrolysis to remodel chromatin and facilitate binding of transcription factors, with resulting increased in gene expression [198, 199]. The ARID proteins have also been implicated in the control of cell growth and differentiation [200, 201]. Thus, decreased ARID2 expression is consistent with the results of methamphetamine withdrawal-induced decreased levels of many transcripts at the delayed time point (see Table 2). In addition to ARID2, another member of the ARID chromatin-remodeling genes, ARID4A, also showed decreased expression at that time point (Fig. 6b). ARID4A possesses an ARID domain, a chromodomain, a Tudor domain, and two repression domains [197, 202]. Chromodomains and Tudor domains regulate binding to methylated lysines in the tails of histones H3 and H4 [203, 204]. ARID4A binds the retinoblastoma protein (pRB) [205, 206], an important regulator of cell proliferation and differentiation [207]. Binding of ARID4A to pRB has been reported to suppress E2F target genes by both HDAC-dependent and HDAC-independent mechanisms [202]. The downregulation of these two ARID transcripts whose protein products are involved in transcription regulation further implicates epigenetic mechanisms in the long-term effects of methamphetamine withdrawal.

Thus, it is of interest that the transcription regulator, LRCH4 (also called LRRN1 or SAP25), a component of the mSin3 co-repressor complex [208, 209] that is used by several classes of transcriptional repressors including MeCP2 [210] and Ikaros [211], is also downregulated after a lengthy withdrawal from methamphetamine. Interestingly, the Ikaros family zinc finger 2 (Helios, IKZF2) is also downregulated at the same time point (see Fig. 6b). Helios is involved in the silencing of IL2 gene in regulatory T cells [212], and its presence in striatal cells [213] suggests that Helios might play a comparable role in the brain immune responses to methamphetamine [43, 214]. In any case, the fact that the levels of several transcripts of proteins that participate in co-repressor complexes are decreased at 1 month after methamphetamine withdrawal suggests that there might be a general depressing effect on transcription at that time, with only a few genes being upregulated after that time interval. It remains to be determined whether the upregulated genes are targets of these co-repressor complexes since the downregulation of transcriptional suppressors would result in their increased transcription.

## Concluding Remarks

In summary, methamphetamine use disorder is a chronic neuropsychiatric disorder that is characterized by a complex clinical course with periods of active drug-taking behaviors filled with bingeing episodes interspersed between drug-free intervals and repeated relapses. Although various neuroimaging studies have identified potential loci for the functional neuroanatomy of its varied clinical presentations, much remains to be done to identify the pathobiological substrates of methamphetamine addiction. It is important to note that human methamphetamine addicts use the drug according to different scheduling patterns and the amount of drug ingested. They also present with a diversity of clinical findings including depression, suicidal ideations, and psychotic symptoms. These clinical observations suggest that the drug might cause differential molecular and neurobiological alterations that produced complex clinical pictures. These statements suggest the need for the development of a diversity of models in which investigators could study the molecular impact of different drug doses that are self-administered by rats. Importantly, similar to the case of other complex neuropsychiatric disorders such as the major affective disorders or schizophrenia, it is very likely that single-gene approaches will fail to provide a comprehensive understanding of the basic neurobiology of drug addiction. Approaches that include genome-wide studies in conjunctions with models that are more representative of the human condition will create better opportunity to clarify the molecular neuropathology of methamphetamine addiction. These approaches promise to help to generate testable hypotheses and ideas that might be translatable to therapeutic approaches. The veracity of this notion is presently being tested in our laboratory by using behavioral models in conjunction with modern molecular techniques.

## Abbreviations

AD: Alzheimer’s disease
AP1: Activating protein 1
ARID: AT-rich interactive domain
ATF: Activating transcription factor
BASP1: Brain abundant signal protein/brain acid soluble protein 1
BDNF: Brain-derived neurotrophic factor
CBP: CREB-binding protein
CDK: Cyclin-dependent kinase
CH: Calponin homology
CNS: Central nervous systems
CREB: Cyclic AMP response element binding
DA: Dopamine
DUSPs: Dual-specificity phosphatases
Egr1: Early growth factor 1
eIF: Eukaryotic translation initiation factor
ELK1: ETS domain-containing protein
ER: Endoplasmic reticulum
ERK: Extracellular signal-regulated kinases
ETS: E-twenty-six domain transcription factor
H3K4me3: Trimethylated lysine 4 of histone 3
HDAC: Histone deacetylase
Helios/IKZF2: Ikaros family zinc finger 2
IEGs: Immediate early genes
JARID1B/KDM5B: Jumonji AT-rich interactive domain 1B/lysine-specific demethylase 5B
KCNC2: Potassium voltage-gated channel, Shaw family
KCNH2: Potassium voltage-gated channel, subfamily H
KLF10: Kruppel-like zinc finger 10
LRCH4: Leucine-rich repeats domain containing 4
MAPK: Mitogen-activated protein kinases
MeCP2: Methyl CpG binding protein 2
NGF: Nerve growth factor
Nr4a1: Nuclear receptor subfamily 4, group A, member 1
PERK: Protein kinase RNA-like endoplasmic reticulum kinase
PIP2: Phosphatidylinositol 4 5-bisphosphate
PKA: Protein kinase A
PKR: RNA-dependent kinase
pRB: Retinoblastoma protein
PTKs: Protein tyrosine kinases
PTPs: Protein tyrosine phosphatases
PTPRE: Protein tyrosine phosphatase receptor, type E
PTPRU: Protein tyrosine phosphatase receptor, type U
R1–R3: Repression domains
TCF: Ternary complex factor
TrkB: Tyrosine kinase receptor, type 2
WT1: Wilms’ tumor suppressor protein 1

## References

[1] Cadet JL, Bisagno V, Milroy CM (2014). Neuropathology of substance use disorders. Acta Neuropathol.

[2] Gonzales R, Mooney L, Rawson RA (2010). The methamphetamine problem in the United States. Annu Rev Public Health.

[3] Krasnova IN, Cadet JL (2009). Methamphetamine toxicity and messengers of death. Brain Res Rev.

[4] Rusyniak DE (2013). Neurologic manifestations of chronic methamphetamine abuse. Psychiatr Clin N Am.

[5] Dean AC, Groman SM, Morales AM, London ED (2013). An evaluation of the evidence that methamphetamine abuse causes cognitive decline in humans. Neuropsychopharmacology.

[6] Henry BL, Minassian A, Perry W (2010). Effect of methamphetamine dependence on everyday functional ability. Addict Behav.

[7] Sadek JR, Vigil O, Grant I, Heaton RK (2007). The impact of neuropsychological functioning and depressed mood on functional complaints in HIV-1 infection and methamphetamine dependence. J Clin Exp Neuropsychol.

[8] Brecht ML, von Mayrhauser C, Anglin MD (2000). Predictors of relapse after treatment for methamphetamine use. J Psychoactive Drugs.

[9] Brecht ML, Greenwell L, von Mayrhauser C, Anglin MD (2006) Two-year outcomes of treatment for methamphetamine use. J Psychoactive Drugs Suppl 3:415–426 10.1080/02791072.2006.10400605 17357533

[10] Cadet JL, Bisagno V (2013). The primacy of cognition in the manifestations of substance use disorders. Front Neurol.

[11] Krasnova IN, Chiflikyan M, Justinova Z, McCoy MT, Ladenheim B, Jayanthi S, Quintero C, Brannock C, Barnes C, Adair JE, Lehrmann E, Kobeissy FH, Gold MS, Becker KG, Goldberg SR, Cadet JL (2013). CREB phosphorylation regulates striatal transcriptional responses in the self-administration model of methamphetamine addiction in the rat. Neurobiol Dis.

[12] Aguilar-Valles A, Vaissiere T, Griggs EM, Mikaelsson MA, Takacs IF, Young EJ, Rumbaugh G, Miller CA (2013). Methamphetamine-associated memory is regulated by a writer and an eraser of permissive histone methylation. Biol Psychiatry.

[13] Cornish JL, Hunt GE, Robins L, McGregor IS (2012). Regional c-Fos and FosB/DeltaFosB expression associated with chronic methamphetamine self-administration and methamphetamine-seeking behavior in rats. Neuroscience.

[14] Martin TA, Jayanthi S, McCoy MT, Brannock C, Ladenheim B, Garrett T, Lehrmann E, Becker KG, Cadet JL (2012). Methamphetamine causes differential alterations in gene expression and patterns of histone acetylation/hypoacetylation in the rat nucleus accumbens. PLoS One.

[15] Jayanthi S, McCoy MT, Chen B, Britt JP, Kourrich S, Yau HJ, Ladenheim B, Krasnova IN, Bonci A, Cadet JL (2013). Methamphetamine downregulates striatal glutamate receptors via diverse epigenetic mechanisms. Biol Psychiatry.

[16] Belin D, Everitt BJ (2008). Cocaine seeking habits depend upon dopamine-dependent serial connectivity linking the ventral with the dorsal striatum. Neuron.

[17] Nestler EJ (2012). Transcriptional mechanisms of drug addiction. Clin Psychopharmacol Neurosci.

[18] Nestler EJ (2014). Epigenetic mechanisms of drug addiction. Neuropharmacology.

[19] Cadet JL, McCoy MT, Cai NS, Krasnova IN, Ladenheim B, Beauvais G, Wilson N, Wood W, Becker KG, Hodges AB (2009). Methamphetamine preconditioning alters midbrain transcriptional responses to methamphetamine-induced injury in the rat striatum. PLoS One.

[20] Kodama M, Akiyama K, Ujike H, Shimizu Y, Tanaka Y, Kuroda S (1998). A robust increase in expression of arc gene, an effector immediate early gene, in the rat brain after acute and chronic methamphetamine administration. Brain Res.

[21] Krasnova IN, Ladenheim B, Hodges AB, Volkow ND, Cadet JL (2011). Chronic methamphetamine administration causes differential regulation of transcription factors in the rat midbrain. PLoS One.

[22] McCoy MT, Jayanthi S, Wulu JA, Beauvais G, Ladenheim B, Martin TA, Krasnova IN, Hodges AB, Cadet JL (2011). Chronic methamphetamine exposure suppresses the striatal expression of members of multiple families of immediate early genes (IEGs) in the rat: normalization by an acute methamphetamine injection. Psychopharmacology (Berl).

[23] Wang JQ, Smith AJ, McGinty JF (1995). A single injection of amphetamine or methamphetamine induces dynamic alterations in c-fos, zif/268 and preprodynorphin messenger RNA expression in rat forebrain. Neuroscience.

[24] Krasnova IN, Justinova Z, Ladenheim B, Jayanthi S, McCoy MT, Barnes C, Warner JE, Goldberg SR, Cadet JL (2010). Methamphetamine self-administration is associated with persistent biochemical alterations in striatal and cortical dopaminergic terminals in the rat. PLoS One.

[25] Smith KS, Graybiel AM (2013). A dual operator view of habitual behavior reflecting cortical and striatal dynamics. Neuron.

[26] Yin HH, Knowlton BJ (2006). The role of the basal ganglia in habit formation. Nat Rev Neurosci.

[27] Graybiel AM (2008). Habits, rituals, and the evaluative brain. Annu Rev Neurosci.

[28] Feng J, Wilkinson M, Liu X, Purushothaman I, Ferguson D, Vialou V, Maze I, Shao N, Kennedy P, Koo J, Dias C, Laitman B, Stockman V, Laplant Q, Cahill M, Nestler EJ, Shen L (2014). Chronic cocaine-regulated epigenomic changes in mouse nucleus accumbens. Genome Biol.

[29] Ferguson D, Koo JW, Feng J, Heller E, Rabkin J, Heshmati M, Renthal W, Neve R, Liu X, Shao N, Sartorelli V, Shen L, Nestler EJ (2013). Essential role of SIRT1 signaling in the nucleus accumbens in cocaine and morphine action. J Neurosci.

[30] McFadden LM, Hadlock GC, Allen SC, Vieira-Brock PL, Stout KA, Ellis JD, Hoonakker AJ, Andrenyak DM, Nielsen SM, Wilkins DG, Hanson GR, Fleckenstein AE (2012). Methamphetamine self-administration causes persistent striatal dopaminergic alterations and mitigates the deficits caused by a subsequent methamphetamine exposure. J Pharmacol Exp Ther.

[31] Schwendt M, Rocha A, See RE, Pacchioni AM, McGinty JF, Kalivas PW (2009). Extended methamphetamine self-administration in rats results in a selective reduction of dopamine transporter levels in the prefrontal cortex and dorsal striatum not accompanied by marked monoaminergic depletion. J Pharmacol Exp Ther.

[32] Schwendt M, Reichel CM, See RE (2012). Extinction-dependent alterations in corticostriatal mGluR2/3 and mGluR7 receptors following chronic methamphetamine self-administration in rats. PLoS One.

[33] McFadden LM, Vieira-Brock PL, Hanson GR, Fleckenstein AE (2014) Methamphetamine self-administration attenuates hippocampal serotonergic deficits: role of brain-derived neurotrophic factor. Int J Neuropsychopharmacol:1–6. doi:10.1017/S1461145714000327 10.1017/S1461145714000327 PMC4074226 24650575

[34] Recinto P, Samant AR, Chavez G, Kim A, Yuan CJ, Soleiman M, Grant Y, Edwards S, Wee S, Koob GF, George O, Mandyam CD (2012). Levels of neural progenitors in the hippocampus predict memory impairment and relapse to drug seeking as a function of excessive methamphetamine self-administration. Neuropsychopharmacology.

[35] Reichel CM, Ramsey LA, Schwendt M, McGinty JF, See RE (2012). Methamphetamine-induced changes in the object recognition memory circuit. Neuropharmacology.

[36] Rogers JL, De Santis S, See RE (2008). Extended methamphetamine self-administration enhances reinstatement of drug seeking and impairs novel object recognition in rats. Psychopharmacology (Berl).

[37] Parsegian A, Glen WB, Lavin A, See RE (2011). Methamphetamine self-administration produces attentional set-shifting deficits and alters prefrontal cortical neurophysiology in rats. Biol Psychiatry.

[38] Jang CG, Whitfield T, Schulteis G, Koob GF, Wee S (2013). A dysphoric-like state during early withdrawal from extended access to methamphetamine self-administration in rats. Psychopharmacology (Berl).

[39] Kitamura O, Wee S, Specio SE, Koob GF, Pulvirenti L (2006). Escalation of methamphetamine self-administration in rats: a dose-effect function. Psychopharmacology (Berl).

[40] Cadet JL, Jayanthi S, McCoy MT, Ladenheim B, Saint-Preux F, Lehrmann E, De S, Becker KG, Brannock C (2013). Genome-wide profiling identifies a subset of methamphetamine (METH)-induced genes associated with METH-induced increased H4K5Ac binding in the rat striatum. BMC Genomics.

[41] Grant KM, LeVan TD, Wells SM, Li M, Stoltenberg SF, Gendelman HE, Carlo G, Bevins RA (2012). Methamphetamine-associated psychosis. J Neuroimmune Pharmacol.

[42] Kohno M, Morales AM, Ghahremani DG, Hellemann G, London ED (2014). Risky decision making, prefrontal cortex, and mesocorticolimbic functional connectivity in methamphetamine dependence. JAMA Psychiatry.

[43] Sekine Y, Ouchi Y, Sugihara G, Takei N, Yoshikawa E, Nakamura K, Iwata Y, Tsuchiya KJ, Suda S, Suzuki K, Kawai M, Takebayashi K, Yamamoto S, Matsuzaki H, Ueki T, Mori N, Gold MS, Cadet JL (2008). Methamphetamine causes microglial activation in the brains of human abusers. J Neurosci.

[44] Volkow ND, Chang L, Wang GJ, Fowler JS, Leonido-Yee M, Franceschi D, Sedler MJ, Gatley SJ, Hitzemann R, Ding YS, Logan J, Wong C, Miller EN (2001). Association of dopamine transporter reduction with psychomotor impairment in methamphetamine abusers. Am J Psychiatry.

[45] Maekawa S, Maekawa M, Hattori S, Nakamura S (1993). Purification and molecular cloning of a novel acidic calmodulin binding protein from rat brain. J Biol Chem.

[46] Mosevitsky MI (2005). Nerve ending "signal" proteins GAP-43, MARCKS, and BASP1. Int Rev Cytol.

[47] Shaw JE, Epand RF, Sinnathamby K, Li Z, Bittman R, Epand RM, Yip CM (2006). Tracking peptide-membrane interactions: insights from in situ coupled confocal-atomic force microscopy imaging of NAP-22 peptide insertion and assembly. J Struct Biol.

[48] Frey D, Laux T, Xu L, Schneider C, Caroni P (2000). Shared and unique roles of CAP23 and GAP43 in actin regulation, neurite outgrowth, and anatomical plasticity. J Cell Biol.

[49] Korshunova I, Caroni P, Kolkova K, Berezin V, Bock E, Walmod PS (2008). Characterization of BASP1-mediated neurite outgrowth. J Neurosci Res.

[50] Carpenter B, Hill KJ, Charalambous M, Wagner KJ, Lahiri D, James DI, Andersen JS, Schumacher V, Royer-Pokora B, Mann M, Ward A, Roberts SG (2004). BASP1 is a transcriptional cosuppressor for the Wilms' tumor suppressor protein WT1. Mol Cell Biol.

[51] Goodfellow SJ, Rebello MR, Toska E, Zeef LA, Rudd SG, Medler KF, Roberts SG (2011). WT1 and its transcriptional cofactor BASP1 redirect the differentiation pathway of an established blood cell line. Biochem J.

[52] Green LM, Wagner KJ, Campbell HA, Addison K, Roberts SG (2009). Dynamic interaction between WT1 and BASP1 in transcriptional regulation during differentiation. Nucleic Acids Res.

[53] Toska E, Campbell HA, Shandilya J, Goodfellow SJ, Shore P, Medler KF, Roberts SG (2012). Repression of transcription by WT1-BASP1 requires the myristoylation of BASP1 and the PIP2-dependent recruitment of histone deacetylase. Cell Rep.

[54] Hollenhorst PC, McIntosh LP, Graves BJ (2011). Genomic and biochemical insights into the specificity of ETS transcription factors. Annu Rev Biochem.

[55] Sharrocks AD (1995). ERK2/p42 MAP kinase stimulates both autonomous and SRF-dependent DNA binding by Elk-1. FEBS Lett.

[56] Yang SH, Whitmarsh AJ, Davis RJ, Sharrocks AD (1998). Differential targeting of MAP kinases to the ETS-domain transcription factor Elk-1. Embo J.

[57] Cahill E, Salery M, Vanhoutte P, Caboche J (2014). Convergence of dopamine and glutamate signaling onto striatal ERK activation in response to drugs of abuse. Front Pharmacol.

[58] Valjent E, Corvol JC, Pages C, Besson MJ, Maldonado R, Caboche J (2000). Involvement of the extracellular signal-regulated kinase cascade for cocaine-rewarding properties. J Neurosci.

[59] Valjent E, Pages C, Herve D, Girault JA, Caboche J (2004). Addictive and non-addictive drugs induce distinct and specific patterns of ERK activation in mouse brain. Eur J Neurosci.

[60] Valjent E, Pascoli V, Svenningsson P, Paul S, Enslen H, Corvol JC, Stipanovich A, Caboche J, Lombroso PJ, Nairn AC, Greengard P, Herve D, Girault JA (2005). Regulation of a protein phosphatase cascade allows convergent dopamine and glutamate signals to activate ERK in the striatum. Proc Natl Acad Sci U S A.

[61] Zheng CF, Guan KL (1993). Properties of MEKs, the kinases that phosphorylate and activate the extracellular signal-regulated kinases. J Biol Chem.

[62] Boulton TG, Nye SH, Robbins DJ, Ip NY, Radziejewska E, Morgenbesser SD, DePinho RA, Panayotatos N, Cobb MH, Yancopoulos GD (1991). ERKs: a family of protein-serine/threonine kinases that are activated and tyrosine phosphorylated in response to insulin and NGF. Cell.

[63] Sgambato V, Vanhoutte P, Pages C, Rogard M, Hipskind R, Besson MJ, Caboche J (1998). In vivo expression and regulation of Elk-1, a target of the extracellular-regulated kinase signaling pathway, in the adult rat brain. J Neurosci.

[64] Odrowaz Z, Sharrocks AD (2012). ELK1 uses different DNA binding modes to regulate functionally distinct classes of target genes. PLoS Genet.

[65] Boros J, Donaldson IJ, O'Donnell A, Odrowaz ZA, Zeef L, Lupien M, Meyer CA, Liu XS, Brown M, Sharrocks AD (2009). Elucidation of the ELK1 target gene network reveals a role in the coordinate regulation of core components of the gene regulation machinery. Genome Res.

[66] O'Donnell A, Odrowaz Z, Sharrocks AD (2012). Immediate-early gene activation by the MAPK pathways: what do and don’t we know?. Biochem Soc Trans.

[67] Sgambato V, Pages C, Rogard M, Besson MJ, Caboche J (1998). Extracellular signal-regulated kinase (ERK) controls immediate early gene induction on corticostriatal stimulation. J Neurosci.

[68] Mao LM, Reusch JM, Fibuch EE, Liu Z, Wang JQ (2013). Amphetamine increases phosphorylation of MAPK/ERK at synaptic sites in the rat striatum and medial prefrontal cortex. Brain Res.

[69] Salzmann J, Marie-Claire C, Le Guen S, Roques BP, Noble F (2003). Importance of ERK activation in behavioral and biochemical effects induced by MDMA in mice. Br J Pharmacol.

[70] Zhao N, Chen Y, Zhu J, Wang L, Cao G, Dang Y, Yan C, Wang J, Chen T (2014). Levo-tetrahydropalmatine attenuates the development and expression of methamphetamine-induced locomotor sensitization and the accompanying activation of ERK in the nucleus accumbens and caudate putamen in mice. Neuroscience.

[71] Besnard A, Bouveyron N, Kappes V, Pascoli V, Pages C, Heck N, Vanhoutte P, Caboche J (2011). Alterations of molecular and behavioral responses to cocaine by selective inhibition of Elk-1 phosphorylation. J Neurosci.

[72] Adams JP, Sweatt JD (2002). Molecular psychology: roles for the ERK MAP kinase cascade in memory. Annu Rev Pharmacol Toxicol.

[73] Johannessen M, Moens U (2007). Multisite phosphorylation of the cAMP response element-binding protein (CREB) by a diversity of protein kinases. Front Biosci.

[74] Davis S, Vanhoutte P, Pages C, Caboche J, Laroche S (2000). The MAPK/ERK cascade targets both Elk-1 and cAMP response element-binding protein to control long-term potentiation-dependent gene expression in the dentate gyrus in vivo. J Neurosci.

[75] Choe ES, McGinty JF (2001). Cyclic AMP and mitogen-activated protein kinases are required for glutamate-dependent cyclic AMP response element binding protein and Elk-1 phosphorylation in the dorsal striatum in vivo. J Neurochem.

[76] Matamales M, Girault JA (2011). Signaling from the cytoplasm to the nucleus in striatal medium-sized spiny neurons. Front Neuroanat.

[77] Shaywitz AJ, Greenberg ME (1999). CREB: a stimulus-induced transcription factor activated by a diverse array of extracellular signals. Annu Rev Biochem.

[78] Janknecht R (2002). The versatile functions of the transcriptional coactivators p300 and CBP and their roles in disease. Histol Histopathol.

[79] Barco A, Patterson SL, Alarcon JM, Gromova P, Mata-Roig M, Morozov A, Kandel ER (2005). Gene expression profiling of facilitated L-LTP in VP16-CREB mice reveals that BDNF is critical for the maintenance of LTP and its synaptic capture. Neuron.

[80] Beaumont TL, Yao B, Shah A, Kapatos G, Loeb JA (2012). Layer-specific CREB target gene induction in human neocortical epilepsy. J Neurosci.

[81] Cadet JL, Jayanthi S, McCoy MT, Beauvais G, Cai NS (2010). Dopamine D1 receptors, regulation of gene expression in the brain, and neurodegeneration. CNS Neurol Disord Drug Targets.

[82] Carlezon WA, Duman RS, Nestler EJ (2005). The many faces of CREB. Trends Neurosci.

[83] Turgeon SM, Pollack AE, Fink JS (1997). Enhanced CREB phosphorylation and changes in c-Fos and FRA expression in striatum accompany amphetamine sensitization. Brain Res.

[84] Xu W, Kasper LH, Lerach S, Jeevan T, Brindle PK (2007). Individual CREB-target genes dictate usage of distinct cAMP-responsive coactivation mechanisms. Embo J.

[85] Alberini CM (2009). Transcription factors in long-term memory and synaptic plasticity. Physiol Rev.

[86] Kandel ER (2012). The molecular biology of memory: cAMP, PKA, CRE, CREB-1, CREB-2, and CPEB. Mol Brain.

[87] Perez-Cadahia B, Drobic B, Davie JR (2011). Activation and function of immediate-early genes in the nervous system. Biochem Cell Biol.

[88] Kim DJ, Roh S, Kim Y, Yoon SJ, Lee HK, Han CS, Kim YK (2005). High concentrations of plasma brain-derived neurotrophic factor in methamphetamine users. Neurosci Lett.

[89] Russo SJ, Mazei-Robison MS, Ables JL, Nestler EJ (2009). Neurotrophic factors and structural plasticity in addiction. Neuropharmacology.

[90] Bykhovskaia M (2011). Synapsin regulation of vesicle organization and functional pools. Semin Cell Dev Biol.

[91] Kwon SE, Chapman ER (2011). Synaptophysin regulates the kinetics of synaptic vesicle endocytosis in central neurons. Neuron.

[92] Greengard P, Valtorta F, Czernik AJ, Benfenati F (1993). Synaptic vesicle phosphoproteins and regulation of synaptic function. Science.

[93] Kao HT, Porton B, Hilfiker S, Stefani G, Pieribone VA, DeSalle R, Greengard P (1999). Molecular evolution of the synapsin gene family. J Exp Zool.

[94] Fornasiero EF, Bonanomi D, Benfenati F, Valtorta F (2010). The role of synapsins in neuronal development. Cell Mol Life Sci.

[95] Hilfiker S, Pieribone VA, Czernik AJ, Kao HT, Augustine GJ, Greengard P (1999). Synapsins as regulators of neurotransmitter release. Philos Trans R Soc Lond B Biol Sci.

[96] Kao HT, Song HJ, Porton B, Ming GL, Hoh J, Abraham M, Czernik AJ, Pieribone VA, Poo MM, Greengard P (2002). A protein kinase A-dependent molecular switch in synapsins regulates neurite outgrowth. Nat Neurosci.

[97] Hosaka M, Hammer RE, Sudhof TC (1999). A phospho-switch controls the dynamic association of synapsins with synaptic vesicles. Neuron.

[98] Menegon A, Bonanomi D, Albertinazzi C, Lotti F, Ferrari G, Kao HT, Benfenati F, Baldelli P, Valtorta F (2006). Protein kinase A-mediated synapsin I phosphorylation is a central modulator of Ca2+-dependent synaptic activity. J Neurosci.

[99] Jedynak JP, Uslaner JM, Esteban JA, Robinson TE (2007). Methamphetamine-induced structural plasticity in the dorsal striatum. Eur J Neurosci.

[100] Rauskolb S, Zagrebelsky M, Dreznjak A, Deogracias R, Matsumoto T, Wiese S, Erne B, Sendtner M, Schaeren-Wiemers N, Korte M, Barde YA (2010). Global deprivation of brain-derived neurotrophic factor in the CNS reveals an area-specific requirement for dendritic growth. J Neurosci.

[101] Soulsby M, Bennett AM (2009). Physiological signaling specificity by protein tyrosine phosphatases. Physiology (Bethesda).

[102] Caunt CJ, Keyse SM (2013). Dual-specificity MAP kinase phosphatases (MKPs): shaping the outcome of MAP kinase signalling. Febs J.

[103] Tonks NK (2013). Protein tyrosine phosphatases–from housekeeping enzymes to master regulators of signal transduction. Febs J.

[104] Patterson KI, Brummer T, O'Brien PM, Daly RJ (2009). Dual-specificity phosphatases: critical regulators with diverse cellular targets. Biochem J.

[105] Muda M, Manning ER, Orth K, Dixon JE (1999). Identification of the human YVH1 protein-tyrosine phosphatase orthologue reveals a novel zinc binding domain essential for in vivo function. J Biol Chem.

[106] Sharda PR, Bonham CA, Mucaki EJ, Butt Z, Vacratsis PO (2009). The dual-specificity phosphatase hYVH1 interacts with Hsp70 and prevents heat-shock-induced cell death. Biochem J.

[107] Bonham CA, Vacratsis PO (2009). Redox regulation of the human dual specificity phosphatase YVH1 through disulfide bond formation. J Biol Chem.

[108] Cadet JL, Sheng P, Ali S, Rothman R, Carlson E, Epstein C (1994). Attenuation of methamphetamine-induced neurotoxicity in copper/zinc superoxide dismutase transgenic mice. J Neurochem.

[109] Mohebiany AN, Nikolaienko RM, Bouyain S, Harroch S (2013). Receptor-type tyrosine phosphatase ligands: looking for the needle in the haystack. Febs J.

[110] Fuchs M, Wang H, Ciossek T, Chen Z, Ullrich A (1998). Differential expression of MAM-subfamily protein tyrosine phosphatases during mouse development. Mech Dev.

[111] Jacobs FM, van der Linden AJ, Wang Y, von Oerthel L, Sul HS, Burbach JP, Smidt MP (2009). Identification of Dlk1, Ptpru and Klhl1 as novel Nurr1 target genes in meso-diencephalic dopamine neurons. Development.

[112] Schepens J, Zeeuwen P, Wieringa B, Hendriks W (1992). Identification and typing of members of the protein-tyrosine phosphatase gene family expressed in mouse brain. Mol Biol Rep.

[113] Sommer L, Rao M, Anderson DJ (1997). RPTP delta and the novel protein tyrosine phosphatase RPTP psi are expressed in restricted regions of the developing central nervous system. Dev Dyn.

[114] Tiran Z, Peretz A, Sines T, Shinder V, Sap J, Attali B, Elson A (2006). Tyrosine phosphatases epsilon and alpha perform specific and overlapping functions in regulation of voltage-gated potassium channels in Schwann cells. Mol Biol Cell.

[115] Toledano-Katchalski H, Kraut J, Sines T, Granot-Attas S, Shohat G, Gil-Henn H, Yung Y, Elson A (2003). Protein tyrosine phosphatase epsilon inhibits signaling by mitogen-activated protein kinases. Mol Cancer Res.

[116] Wang H, Lian Z, Lerch MM, Chen Z, Xie W, Ullrich A (1996). Characterization of PCP-2, a novel receptor protein tyrosine phosphatase of the MAM domain family. Oncogene.

[117] Yan HX, He YQ, Dong H, Zhang P, Zeng JZ, Cao HF, Wu MC, Wang HY (2002). Physical and functional interaction between receptor-like protein tyrosine phosphatase PCP-2 and beta-catenin. Biochemistry.

[118] MacDonald BT, Tamai K, He X (2009). Wnt/beta-catenin signaling: components, mechanisms, and diseases. Dev Cell.

[119] Yan HX, Yang W, Zhang R, Chen L, Tang L, Zhai B, Liu SQ, Cao HF, Man XB, Wu HP, Wu MC, Wang HY (2006). Protein-tyrosine phosphatase PCP-2 inhibits beta-catenin signaling and increases E-cadherin-dependent cell adhesion. J Biol Chem.

[120] Badde A, Schulte D (2008). A role for receptor protein tyrosine phosphatase lambda in midbrain development. J Neurosci.

[121] Aerne B, Ish-Horowicz D (2004). Receptor tyrosine phosphatase psi is required for Delta/Notch signalling and cyclic gene expression in the presomitic mesoderm. Development.

[122] Badde A, Bumsted-O'Brien KM, Schulte D (2005). Chick receptor protein tyrosine phosphatase lambda/psi (cRPTPlambda/cRPTPpsi) is dynamically expressed at the midbrain-hindbrain boundary and in the embryonic neural retina. Gene Expr Patterns.

[123] Ben Hamida S, Darcq E, Wang J, Wu S, Phamluong K, Kharazia V, Ron D (2013). Protein tyrosine phosphatase alpha in the dorsomedial striatum promotes excessive ethanol-drinking behaviors. J Neurosci.

[124] McConnell BB, Yang VW (2010). Mammalian Kruppel-like factors in health and diseases. Physiol Rev.

[125] Song KD, Kim DJ, Lee JE, Yun CH, Lee WK (2012). KLF10, transforming growth factor-beta-inducible early gene 1, acts as a tumor suppressor. Biochem Biophys Res Commun.

[126] Cook T, Gebelein B, Belal M, Mesa K, Urrutia R (1999). Three conserved transcriptional repressor domains are a defining feature of the TIEG subfamily of Sp1-like zinc finger proteins. J Biol Chem.

[127] Zhang JS, Moncrieffe MC, Kaczynski J, Ellenrieder V, Prendergast FG, Urrutia R (2001). A conserved alpha-helical motif mediates the interaction of Sp1-like transcriptional repressors with the corepressor mSin3A. Mol Cell Biol.

[128] Kim J, Shin S, Subramaniam M, Bruinsma E, Kim TD, Hawse JR, Spelsberg TC, Janknecht R (2010). Histone demethylase JARID1B/KDM5B is a corepressor of TIEG1/KLF10. Biochem Biophys Res Commun.

[129] Upadhyay AK, Cheng X (2011). Dynamics of histone lysine methylation: structures of methyl writers and erasers. Prog Drug Res.

[130] Martin C, Zhang Y (2005). The diverse functions of histone lysine methylation. Nat Rev Mol Cell Biol.

[131] Ng SS, Yue WW, Oppermann U, Klose RJ (2009). Dynamic protein methylation in chromatin biology. Cell Mol Life Sci.

[132] Newton TF, De La Garza R, Kalechstein AD, Tziortzis D, Jacobsen CA (2009). Theories of addiction: methamphetamine users' explanations for continuing drug use and relapse. Am J Addict.

[133] Shepard JD, Bossert JM, Liu SY, Shaham Y (2004). The anxiogenic drug yohimbine reinstates methamphetamine seeking in a rat model of drug relapse. Biol Psychiatry.

[134] Pickens CL, Airavaara M, Theberge F, Fanous S, Hope BT, Shaham Y (2011). Neurobiology of the incubation of drug craving. Trends Neurosci.

[135] Krasnova IN, Marchant NJ, Ladenheim B, McCoy MT, Panlilio LV, Bossert JM, Shaham Y, Cadet JL (2014). Incubation of methamphetamine and palatable food craving after punishment-induced abstinence. Neuropsychopharmacology.

[136] Adachi M, Monteggia LM (2014). Decoding transcriptional repressor complexes in the adult central nervous system. Neuropharmacology.

[137] Kelly RD, Cowley SM (2013). The physiological roles of histone deacetylase (HDAC) 1 and 2: complex co-stars with multiple leading parts. Biochem Soc Trans.

[138] Graff J, Tsai LH (2013). Histone acetylation: molecular mnemonics on the chromatin. Nat Rev Neurosci.

[139] Guan JS, Haggarty SJ, Giacometti E, Dannenberg JH, Joseph N, Gao J, Nieland TJ, Zhou Y, Wang X, Mazitschek R, Bradner JE, DePinho RA, Jaenisch R, Tsai LH (2009). HDAC2 negatively regulates memory formation and synaptic plasticity. Nature.

[140] Kim MS, Akhtar MW, Adachi M, Mahgoub M, Bassel-Duby R, Kavalali ET, Olson EN, Monteggia LM (2012). An essential role for histone deacetylase 4 in synaptic plasticity and memory formation. J Neurosci.

[141] Vecsey CG, Hawk JD, Lattal KM, Stein JM, Fabian SA, Attner MA, Cabrera SM, McDonough CB, Brindle PK, Abel T, Wood MA (2007). Histone deacetylase inhibitors enhance memory and synaptic plasticity via CREB:CBP-dependent transcriptional activation. J Neurosci.

[142] Itzhak Y, Liddie S, Anderson KL (2013). Sodium butyrate-induced histone acetylation strengthens the expression of cocaine-associated contextual memory. Neurobiol Learn Mem.

[143] Kennedy PJ, Feng J, Robison AJ, Maze I, Badimon A, Mouzon E, Chaudhury D, Damez-Werno DM, Haggarty SJ, Han MH, Bassel-Duby R, Olson EN, Nestler EJ (2013). Class I HDAC inhibition blocks cocaine-induced plasticity by targeted changes in histone methylation. Nat Neurosci.

[144] Malvaez M, McQuown SC, Rogge GA, Astarabadi M, Jacques V, Carreiro S, Rusche JR, Wood MA (2013). HDAC3-selective inhibitor enhances extinction of cocaine-seeking behavior in a persistent manner. Proc Natl Acad Sci U S A.

[145] Malvaez M, Sanchis-Segura C, Vo D, Lattal KM, Wood MA (2010). Modulation of chromatin modification facilitates extinction of cocaine-induced conditioned place preference. Biol Psychiatry.

[146] Romieu P, Host L, Gobaille S, Sandner G, Aunis D, Zwiller J (2008). Histone deacetylase inhibitors decrease cocaine but not sucrose self-administration in rats. J Neurosci.

[147] Sanchis-Segura C, Lopez-Atalaya JP, Barco A (2009). Selective boosting of transcriptional and behavioral responses to drugs of abuse by histone deacetylase inhibition. Neuropsychopharmacology.

[148] Costa-Mattioli M, Sossin WS, Klann E, Sonenberg N (2009). Translational control of long-lasting synaptic plasticity and memory. Neuron.

[149] Trinh MA, Klann E (2013). Translational control by eIF2alpha kinases in long-lasting synaptic plasticity and long-term memory. Neurobiol Learn Mem.

[150] Agnihotri NT, Hawkins RD, Kandel ER, Kentros C (2004). The long-term stability of new hippocampal place fields requires new protein synthesis. Proc Natl Acad Sci U S A.

[151] Kandel ER (2001). The molecular biology of memory storage: a dialogue between genes and synapses. Science.

[152] Donnelly N, Gorman AM, Gupta S, Samali A (2013). The eIF2alpha kinases: their structures and functions. Cell Mol Life Sci.

[153] Sachs AB, Sarnow P, Hentze MW (1997). Starting at the beginning, middle, and end: translation initiation in eukaryotes. Cell.

[154] Lackner DH, Schmidt MW, Wu S, Wolf DA, Bahler J (2012). Regulation of transcriptome, translation, and proteome in response to environmental stress in fission yeast. Genome Biol.

[155] Jayanthi S, Deng X, Noailles PA, Ladenheim B, Cadet JL (2004). Methamphetamine induces neuronal apoptosis via cross-talks between endoplasmic reticulum and mitochondria-dependent death cascades. Faseb J.

[156] Jayanthi S, McCoy MT, Beauvais G, Ladenheim B, Gilmore K, Wood W, Becker K, Cadet JL (2009). Methamphetamine induces dopamine D1 receptor-dependent endoplasmic reticulum stress-related molecular events in the rat striatum. PLoS One.

[157] Jahn H (2013). Memory loss in Alzheimer’s disease. Dialogues Clin Neurosci.

[158] Perl DP (2010). Neuropathology of Alzheimer’s disease. Mt Sinai J Med.

[159] Peel AL, Bredesen DE (2003). Activation of the cell stress kinase PKR in Alzheimer’s disease and human amyloid precursor protein transgenic mice. Neurobiol Dis.

[160] Page G, Rioux Bilan A, Ingrand S, Lafay-Chebassier C, Pain S, Perault Pochat MC, Bouras C, Bayer T, Hugon J (2006). Activated double-stranded RNA-dependent protein kinase and neuronal death in models of Alzheimer's disease. Neuroscience.

[161] Sadler AJ, Williams BR (2007). Structure and function of the protein kinase R. Curr Top Microbiol Immunol.

[162] de Haro C, Mendez R, Santoyo J (1996). The eIF-2alpha kinases and the control of protein synthesis. Faseb J.

[163] Harding HP, Zhang Y, Ron D (1999). Protein translation and folding are coupled by an endoplasmic-reticulum-resident kinase. Nature.

[164] Ho YS, Yang X, Lau JC, Hung CH, Wuwongse S, Zhang Q, Wang J, Baum L, So KF, Chang RC (2012). Endoplasmic reticulum stress induces tau pathology and forms a vicious cycle: implication in Alzheimer's disease pathogenesis. J Alzheimers Dis.

[165] Ma T, Trinh MA, Wexler AJ, Bourbon C, Gatti E, Pierre P, Cavener DR, Klann E (2013). Suppression of eIF2alpha kinases alleviates Alzheimer's disease-related plasticity and memory deficits. Nat Neurosci.

[166] Dmitriev SE, Terenin IM, Andreev DE, Ivanov PA, Dunaevsky JE, Merrick WC, Shatsky IN (2010). GTP-independent tRNA delivery to the ribosomal P-site by a novel eukaryotic translation factor. J Biol Chem.

[167] Yamada T, Nakao S, Osada S, Imagawa M, Nishihara T (1995). Sequence analysis of the rat jun-D gene. Gene.

[168] Short JD, Pfarr CM (2002). Translational regulation of the JunD messenger RNA. J Biol Chem.

[169] Hirai SI, Ryseck RP, Mechta F, Bravo R, Yaniv M (1989). Characterization of junD: a new member of the jun proto-oncogene family. Embo J.

[170] Ryder K, Lanahan A, Perez-Albuerne E, Nathans D (1989). jun-D: a third member of the jun gene family. Proc Natl Acad Sci U S A.

[171] Hernandez JM, Floyd DH, Weilbaecher KN, Green PL, Boris-Lawrie K (2008). Multiple facets of junD gene expression are atypical among AP-1 family members. Oncogene.

[172] Vinson C, Myakishev M, Acharya A, Mir AA, Moll JR, Bonovich M (2002). Classification of human B-ZIP proteins based on dimerization properties. Mol Cell Biol.

[173] Shaulian E, Karin M (2002). AP-1 as a regulator of cell life and death. Nat Cell Biol.

[174] Wang Y, Cesena TI, Ohnishi Y, Burger-Caplan R, Lam V, Kirchhoff PD, Larsen SD, Larsen MJ, Nestler EJ, Rudenko G (2012). Small molecule screening identifies regulators of the transcription factor DeltaFosB. ACS Chem Neurosci.

[175] Angel P, Karin M (1991). The role of Jun, Fos and the AP-1 complex in cell-proliferation and transformation. Biochim Biophys Acta.

[176] Karin M, Liu Z, Zandi E (1997). AP-1 function and regulation. Curr Opin Cell Biol.

[177] Hess J, Angel P, Schorpp-Kistner M (2004). AP-1 subunits: quarrel and harmony among siblings. J Cell Sci.

[178] Pfarr CM, Mechta F, Spyrou G, Lallemand D, Carillo S, Yaniv M (1994). Mouse JunD negatively regulates fibroblast growth and antagonizes transformation by ras. Cell.

[179] Weitzman JB, Fiette L, Matsuo K, Yaniv M (2000). JunD protects cells from p53-dependent senescence and apoptosis. Mol Cell.

[180] Gerald D, Berra E, Frapart YM, Chan DA, Giaccia AJ, Mansuy D, Pouyssegur J, Yaniv M, Mechta-Grigoriou F (2004). JunD reduces tumor angiogenesis by protecting cells from oxidative stress. Cell.

[181] Hull RP, Srivastava PK, D'Souza Z, Atanur SS, Mechta-Grigoriou F, Game L, Petretto E, Cook HT, Aitman TJ, Behmoaras J (2013). Combined ChIP-Seq and transcriptome analysis identifies AP-1/JunD as a primary regulator of oxidative stress and IL-1beta synthesis in macrophages. BMC Genomics.

[182] Smart DE, Vincent KJ, Arthur MJ, Eickelberg O, Castellazzi M, Mann J, Mann DA (2001). JunD regulates transcription of the tissue inhibitor of metalloproteinases-1 and interleukin-6 genes in activated hepatic stellate cells. J Biol Chem.

[183] Srivastava PK, Hull RP, Behmoaras J, Petretto E, Aitman TJ (2013). JunD/AP1 regulatory network analysis during macrophage activation in a rat model of crescentic glomerulonephritis. BMC Syst Biol.

[184] Yoon JK, Lau LF (1994). Involvement of JunD in transcriptional activation of the orphan receptor gene nur77 by nerve growth factor and membrane depolarization in PC12 cells. Mol Cell Biol.

[185] Boss V, Roback JD, Young AN, Roback LJ, Weisenhorn DM, Medina-Flores R, Wainer BH (2001). Nerve growth factor, but not epidermal growth factor, increases Fra-2 expression and alters Fra-2/JunD binding to AP-1 and CREB binding elements in pheochromocytoma (PC12) cells. J Neurosci.

[186] Rosenberger SF, Finch JS, Gupta A, Bowden GT (1999). Extracellular signal-regulated kinase 1/2-mediated phosphorylation of JunD and FosB is required for okadaic acid-induced activator protein 1 activation. J Biol Chem.

[187] Lidwell K, Griffiths R (2000). Possible role for the FosB/JunD AP-1 transcription factor complex in glutamate-mediated excitotoxicity in cultured cerebellar granule cells. J Neurosci Res.

[188] Kobierski LA, Chu HM, Tan Y, Comb MJ (1991). cAMP-dependent regulation of proenkephalin by JunD and JunB: positive and negative effects of AP-1 proteins. Proc Natl Acad Sci U S A.

[189] Herdegen T, Kovary K, Buhl A, Bravo R, Zimmermann M, Gass P (1995). Basal expression of the inducible transcription factors c-Jun, JunB, JunD, c-Fos, FosB, and Krox-24 in the adult rat brain. J Comp Neurol.

[190] Herdegen T, Waetzig V (2001). AP-1 proteins in the adult brain: facts and fiction about effectors of neuroprotection and neurodegeneration. Oncogene.

[191] Mellstrom B, Achaval M, Montero D, Naranjo JR, Sassone-Corsi P (1991). Differential expression of the jun family members in rat brain. Oncogene.

[192] Mohrmann L, Langenberg K, Krijgsveld J, Kal AJ, Heck AJ, Verrijzer CP (2004). Differential targeting of two distinct SWI/SNF-related Drosophila chromatin-remodeling complexes. Mol Cell Biol.

[193] Yan Z, Cui K, Murray DM, Ling C, Xue Y, Gerstein A, Parsons R, Zhao K, Wang W (2005). PBAF chromatin-remodeling complex requires a novel specificity subunit, BAF200, to regulate expression of selective interferon-responsive genes. Genes Dev.

[194] Wu JI, Lessard J, Crabtree GR (2009). Understanding the words of chromatin regulation. Cell.

[195] Mohrmann L, Verrijzer CP (2005). Composition and functional specificity of SWI2/SNF2 class chromatin remodeling complexes. Biochim Biophys Acta.

[196] Patsialou A, Wilsker D, Moran E (2005). DNA-binding properties of ARID family proteins. Nucleic Acids Res.

[197] Wilsker D, Probst L, Wain HM, Maltais L, Tucker PW, Moran E (2005). Nomenclature of the ARID family of DNA-binding proteins. Genomics.

[198] Xu F, Flowers S, Moran E (2012). Essential role of ARID2 protein-containing SWI/SNF complex in tissue-specific gene expression. J Biol Chem.

[199] Racki LR, Narlikar GJ (2008). ATP-dependent chromatin remodeling enzymes: two heads are not better, just different. Curr Opin Genet Dev.

[200] Wilsker D, Patsialou A, Dallas PB, Moran E (2002). ARID proteins: a diverse family of DNA binding proteins implicated in the control of cell growth, differentiation, and development. Cell Growth Differ.

[201] Takeuchi T, Watanabe Y, Takano-Shimizu T, Kondo S (2006). Roles of jumonji and jumonji family genes in chromatin regulation and development. Dev Dyn.

[202] Lai A, Lee JM, Yang WM, DeCaprio JA, Kaelin WG, Seto E, Branton PE (1999). RBP1 recruits both histone deacetylase-dependent and -independent repression activities to retinoblastoma family proteins. Mol Cell Biol.

[203] Yap KL, Zhou MM (2011). Structure and mechanisms of lysine methylation recognition by the chromodomain in gene transcription. Biochemistry.

[204] Huang Y, Fang J, Bedford MT, Zhang Y, Xu RM (2006). Recognition of histone H3 lysine-4 methylation by the double tudor domain of JMJD2A. Science.

[205] Chittenden T, Livingston DM, Kaelin WG (1991). The T/E1A-binding domain of the retinoblastoma product can interact selectively with a sequence-specific DNA-binding protein. Cell.

[206] Defeo-Jones D, Huang PS, Jones RE, Haskell KM, Vuocolo GA, Hanobik MG, Huber HE, Oliff A (1991). Cloning of cDNAs for cellular proteins that bind to the retinoblastoma gene product. Nature.

[207] Manning AL, Dyson NJ (2012). RB: mitotic implications of a tumour suppressor. Nat Rev Cancer.

[208] Sahu SC, Swanson KA, Kang RS, Huang K, Brubaker K, Ratcliff K, Radhakrishnan I (2008). Conserved themes in target recognition by the PAH1 and PAH2 domains of the Sin3 transcriptional corepressor. J Mol Biol.

[209] Shiio Y, Rose DW, Aur R, Donohoe S, Aebersold R, Eisenman RN (2006). Identification and characterization of SAP25, a novel component of the mSin3 corepressor complex. Mol Cell Biol.

[210] Nan X, Ng HH, Johnson CA, Laherty CD, Turner BM, Eisenman RN, Bird A (1998). Transcriptional repression by the methyl-CpG-binding protein MeCP2 involves a histone deacetylase complex. Nature.

[211] Koipally J, Renold A, Kim J, Georgopoulos K (1999). Repression by Ikaros and Aiolos is mediated through histone deacetylase complexes. Embo J.

[212] Baine I, Basu S, Ames R, Sellers RS, Macian F (2013). Helios induces epigenetic silencing of IL2 gene expression in regulatory T cells. J Immunol.

[213] Martin-Ibanez R, Crespo E, Esgleas M, Urban N, Wang B, Waclaw R, Georgopoulos K, Martinez S, Campbell K, Vicario-Abejon C, Alberch J, Chan S, Kastner P, Rubenstein JL, Canals JM (2012). Helios transcription factor expression depends on Gsx2 and Dlx1&2 function in developing striatal matrix neurons. Stem Cells Dev.

[214] Shah A, Silverstein PS, Singh DP, Kumar A (2012). Involvement of metabotropic glutamate receptor 5, AKT/PI3K signaling and NF-kappaB pathway in methamphetamine-mediated increase in IL-6 and IL-8 expression in astrocytes. J Neuroinflammation.

